# SAMPL is a high-throughput solution to study unconstrained vertical behavior in small animals

**DOI:** 10.1016/j.celrep.2023.112573

**Published:** 2023-06-01

**Authors:** Yunlu Zhu, Franziska Auer, Hannah Gelnaw, Samantha N. Davis, Kyla R. Hamling, Christina E. May, Hassan Ahamed, Niels Ringstad, Katherine I. Nagel, David Schoppik

**Affiliations:** 1 Department of Otolaryngology, New York University Grossman School of Medicine, New York, NY 10016, USA; 2 The Neuroscience Institute, New York University Grossman School of Medicine, New York, NY 10016, USA; 3 Department of Neuroscience & Physiology, New York University Grossman School of Medicine, New York, NY 10016, USA; 4 Department of Cell Biology, Skirball Institute of Biomolecular Medicine, New York University Grossman School of Medicine, New York, NY 10016, USA; 5 Lead contact

## Abstract

Balance and movement are impaired in many neurological disorders. Recent advances in behavioral monitoring provide unprecedented access to posture and locomotor kinematics but without the throughput and scalability necessary to screen candidate genes/potential therapeutics. Here, we present a scalable apparatus to measure posture and locomotion (SAMPL). SAMPL includes extensible hardware and open-source software with real-time processing and can acquire data from *D. melanogaster, C. elegans*, and *D. rerio* as they move vertically. Using SAMPL, we define how zebrafish balance as they navigate vertically and discover small but systematic variations among kinematic parameters between genetic backgrounds. We demonstrate SAMPL’s ability to resolve differences in posture and navigation as a function of effect size and data gathered, providing key data for screens. SAMPL is therefore both a tool to model balance and locomotor disorders and an exemplar of how to scale apparatus to support screens.

## In brief

Zhu et al. present SAMPL, a low-cost, open-source tool for measuring posture and vertical locomotion in small animals such as flies, worms, and larval fish in real time. SAMPL’s high resolution, scalability, and throughput make it a promising tool for screening modifiers of balance and locomotion.

## Graphical abstract

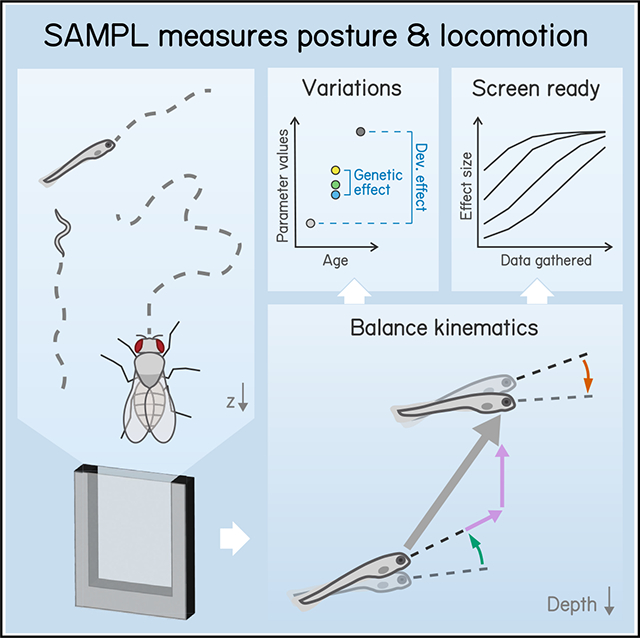

## INTRODUCTION

Measuring posture and locomotion is key to understanding nervous system function and evaluating potential treatments for disease—particularly neurological disorders.^1^ Behavioral screening is a fundamental part of both basic and translational approaches to disease.^2,3^ For screens, measuring behavior from large numbers of animals is necessary to differentiate individual variation^4^ from changes seen in disease models and/or improvement following treatment.^5,6^ The demand for such high-throughput measurements comes at a cost: often, measurements that require high resolution—such as posture—are limited. Modern machine learning algorithms and inexpensive videographic/computing hardware have automated measurements of posture and kinematics^7–9^ and illuminated our understanding of animal behavior.^10–12^ We sought to combine videographic analysis of posture and vertical locomotion with the scalability amenable to screening.

Over the past decade, we have studied posture and locomotion using the larval zebrafish as a model. Neural architecture is highly conserved across vertebrates, making larval zebrafish an excellent model to understand the underpinnings of locomotion^13,14^ and balance.^15^ For our studies, we developed an apparatus/analysis pipeline to measure the statistics of posture in the pitch (nose-up/nose-down) axis and locomotion as larvae swam freely in depth. We discovered that larvae learn to time their movements to facilitate balance,^16^ that larvae modulate the kinematics of swimming to correct posture,^17^ and that larvae engage their pectoral fins to climb efficiently,^18^ and we implicated different neuronal circuits in each of these behaviors. While informative, data collection was slow (months) on small numbers (<5) of apparatus. Increasing throughput remains a challenge common to laboratories that develop tools to measure behavior.

To meet the needs of scalability, resolution, and extensibility, we developed SAMPL (scalable apparatus to measure posture and locomotion): a low-cost, open-source solution that measures posture and vertical locomotion in real time in small animals. Further, we provide a turnkey analysis pipeline to measure larval zebrafish balance behavior. We begin with a brief treatment of the hardware and software; a detailed design guide and assembly and operating instructions are included as appendices in the STAR Methods. Next, we use SAMPL to measure unconstrained vertical locomotion in two common invertebrate models: flies (*Drosophila melanogaster*) and worms (*Caenorhabditis elegans*), as well as a small-model vertebrate, the larval zebrafish (*Danio rerio*). To illustrate SAMPL’s capabilities, we parameterize a dataset focused on behaviors that larval zebrafish perform as they stabilize posture and navigate (i.e., climb/dive) in the water column. Our dataset represents 2 weeks’ worth of data collection and allowed us to detail variation in postural/locomotor behaviors. By measuring behavior across different genetic backgrounds and development, we report two findings. First, variation in posture/locomotion is systematic across genotype, and second, the scale of variation in behavior across development is much larger than background genetic variation. We use these data to simulate the resolving power for each behavioral parameter as a function of data gathered—foundational information to rigorously assay the effects of candidate genes or small molecules on posture or locomotion. SAMPL thus offers a straightforward way to gather data from small animals and a turnkey solution to screen for balance and vertical locomotion in larval zebrafish. More broadly, SAMPL offers a template for laboratories looking to scale their own behavioral apparatus to achieve the throughput necessary for screens. SAMPL will thus facilitate reproducible studies of postural and locomotor behaviors in both health and disease, addressing unmet needs in treating neurological disorders, particularly with balance symptoms.^19^

## RESULTS

### SAMPL hardware and software overview

**Figure 1.**
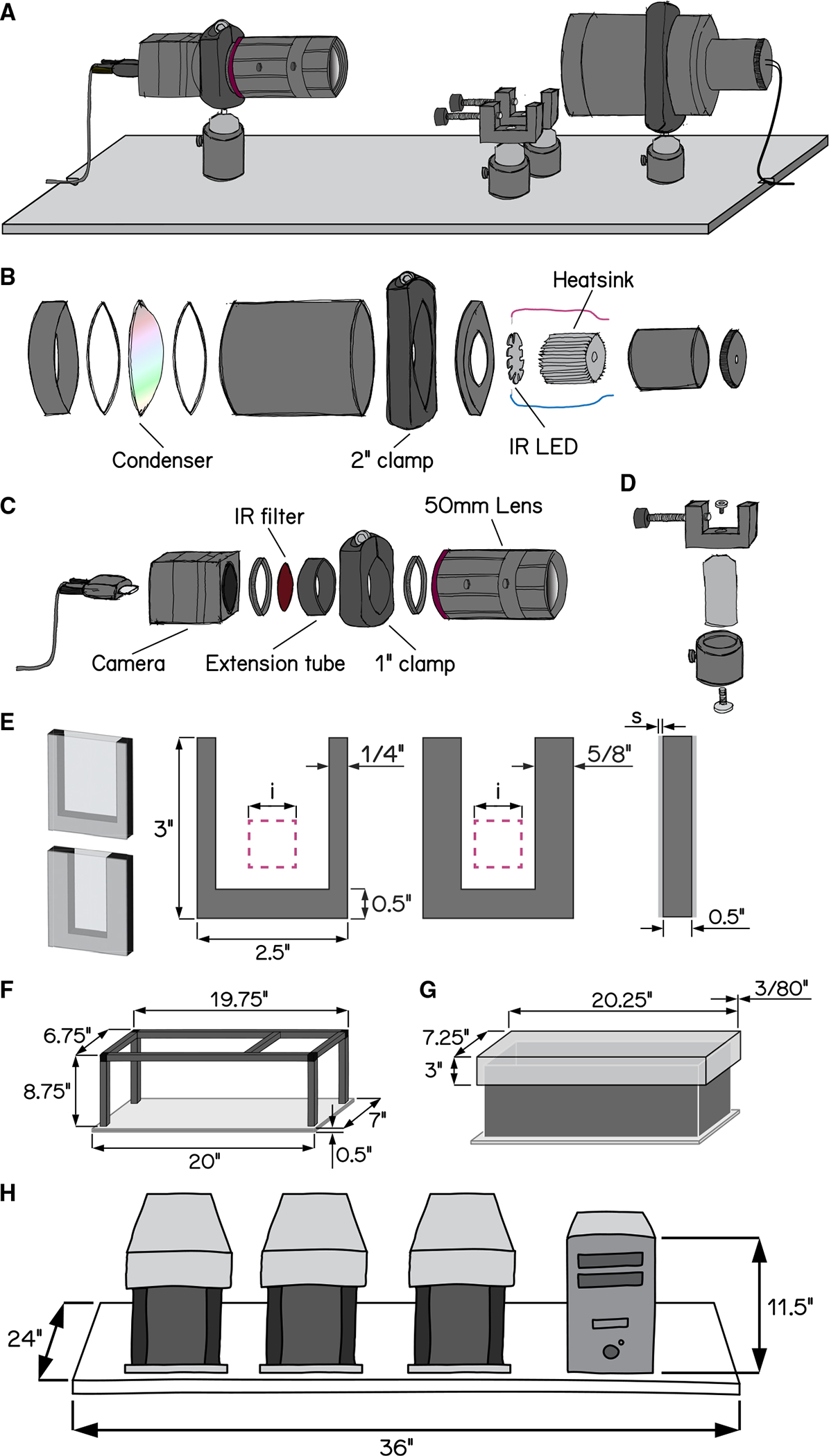
Schematic illustrations of SAMPL hardware design (A) Overview of the apparatus without aluminum rails, side panels, and the top panel. Equipment modules mounted on the breadboard are, from left to right, IR camera and lens, chamber holders, and IR illumination module. (B) Exploded-view drawing of the IR illumination module. (C) Exploded-view drawing of the camera and lens module. (D) Exploded-view drawing of a chamber holder. (E) Design of fish chambers. From left to right: 3D illustration of a standard chamber (top) and a narrow chamber (bottom), front view of the U-shaped acrylic middle piece for the chambers, and side view of the chamber. Pink squares illustrate the recording field of view. i = 20 mm; s = 1.5 mm. (F) Dimensions of the apparatus frame and breadboard. (G) Design and dimensions of the apparatus lid. (H) Schematic illustration of a set of three SAMPL apparatus and a small-form-factor computer case on a 24″ × 36″ shelf. See also Figure S1, Table S1, and Video S1.

To measure posture with the throughput necessary for genetic and drug screens, we deployed SAMPL, a real-time videographic system (Figure 1A) that records small-animal behavior in the vertical axis. Below we briefly describe the hardware and software that comprise SAMPL. SAMPL’s hardware consists of three simple modules: an infrared (IR) illumination module (Figure 1B), a camera-lens module (Figure 1C), and two clamps to hold fish chambers (Figure 1D). All three modules are mounted directly (Figure 1A) onto an aluminum breadboard (Figure S1A), and a light-tight enclosure covers the entire apparatus to permit individual control of lighting (Figures 1F and 1G). Details of hardware and software design can be found in Notes S1 and S2. A complete parts list is in Table S1, hardware assembly instructions are in Note S3, and a stop-motion video of assembly is provided as Video S1.

The IR module illuminates the arena from behind. It is optimized to fulfill four criteria: (1) high image quality, (2) a large area for imaging, (3) imperceptible illumination, and (4) ample heat dissipation. We used a 940 nm “star”-style LED as our source of IR illumination and developed a simple illumination module to diffuse IR light across a 50 mm circle (Figure 1B). For heat management, each LED was mounted to a small heat sink (Figure 1B). This setup allows us to power three illumination modules in series using a single LED driver.

The second module captures videographic data. It consists of a camera and lens optimized for speed, resolution, compactness, and affordability. The camera hardware satisfies the following demands: (1) large pixel size with low noise, allowing for high dynamic range/signal-to-noise ratio, (2) sufficient resolution to resolve subtle changes to animal posture, (3) an interface with sufficient bandwidth for data transfer, and (4) availability. The lens achieves (1) close focus; (2) sufficient depth of field to cover the entire depth of the imaging arena; (3) high image quality; (4) compact size; (5) high IR transmission rate; and (6) ease of integrating an IR-pass filter. We adapted a 50 mm IR-optimized lens by placing a 0.3″ extension tube between the lens and the camera to achieve higher magnification ratio with minimum working distance. The space between camera adapters and the extension tube allows us to fit a 25 mm IR-pass filter; the extension tube gives a mount point to connect the module to the base (Figure 1A). Using this camera-lens module, we image an area ~400 mm^2^ (Figure 1E, pink square) at 166 Hz with 1,200 × 1,216 pixels at a focal distance of ~24 cm.

The final module is a rectangular arena optimized for vertical locomotion (i.e., parallel to the focal plane). By design, the chamber size is larger than the imaging area, allowing stochastic sampling of freely behaving animals in a large enough arena. The bottom of the chamber is below the field of view so that animals sitting at the bottom will not be recorded. We assembled custom-fabricated chambers from laser-cut acrylic by cementing transparent front and back sides to a U-shaped piece that forms the narrower sides (Figure 1E). We designed two types of chambers with different inner widths to adapt to the needs of different experiments: a wider standard chamber optimized for larger groups of animals and a narrower chamber for 1–3 animals (Figure 1E). Chambers can be easily dropped into the holders (Figure 1D) from the top of the behavior box and secured in place for recording.

SAMPL includes a complete suite of open-source software for acquisition/real-time extraction of data (source and compiled executables provided). Acquisition consists of a graphical user interface written in LabView that analyzes video in real time to isolate an animal’s location and orientation, with the ability to save raw video for further offline analysis. The real-time processing algorithm consists of (1) background subtraction; (2) noise thresholding; (3) rejection of frames without an animal or with >1 animal in view; (4) size and intensity criteria to identify two distinct animal parts, usually the body and the head; and (5) image processing to extract location and body orientation relative to the horizon. Data about location and orientation are saved to a text file, metadata about the experiment are saved to a separate text file, and, optionally, a video is saved as an AVI file.

SAMPL’s modules and software were designed to scale, minimizing footprint and experimenter time. We multiplex the apparatus, providing three distinct compiled applications designed to run simultaneously on one computer to reduce cost/footprint. A set of three SAMPL apparatus and a computer case fit on one 24″ × 36″ shelf (Figure 1H). One SAMPL “rack” consists of four such shelves (81.5” high) and costs ~$40,000–45,000 (December 2022, before volume discounts). In our laboratory, trained experimenters can load such a rack for a typical 48 h experiment in 30 min. Taken together, SAMPL’s design is ideal to efficiently gather data describing posture and vertical locomotion.

### SAMPL validation: Different small animals

**Figure 2.**
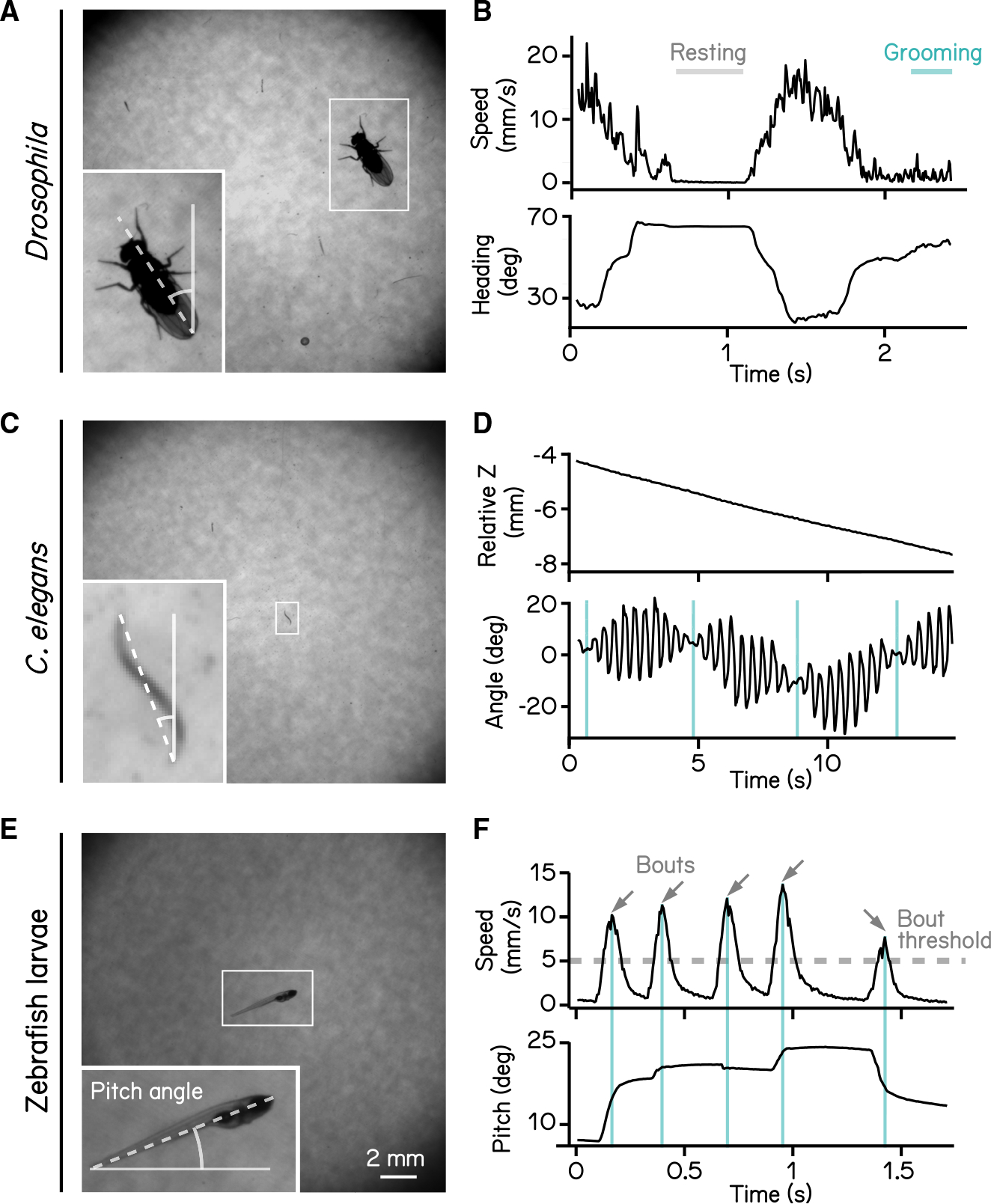
High-definition recording and measurement of animal locomotion using SAMPL (A) Example of a recorded frame with a *Drosophila melanogaster* (white box) in the SAMPL apparatus. Dashed line indicates heading of the fly relative to vertical up (north). Imaging was performed at 166 Hz with 1,200 × 1,216 pixels. Same as follows. (B) Example of an epoch of a walking fly. Walking speed and heading are plotted as a function of time. Gray and cyan lines mark resting and grooming periods, respectively (Video S2). (C) Example of a recorded frame with a *Caenorhabditis elegans* (white box) in the SAMPL apparatus. Dashed line indicates approximated angle of the worm relative to vertical. (D) Example of an epoch of a swimming worm. z position and approximated angle are plotted as a function of time. Cyan vertical lines label the time when the plane of movement is perpendicular to the imaging plane (Video S2). (E) Example of a recorded frame with a 7 dpf *Danio rerio* larva (white box) in the SAMPL apparatus. Pitch angle is determined as the angle of the trunk of the fish (dashed line) relative to horizontal. Positive pitch indicates nose-up posture, whereas negative pitch represents nose-down posture. (F) Example of an epoch containing multiple swim bouts (arrows). Swim speed and pitch angles are plotted as a function of time. Dashed line marks the 5 mm/s threshold for bout detection. Cyan vertical lines label time of the peak speed for each bout. See also Table S2 and Videos S2 and S3.

SAMPL is well suited to collect data from a wide range of small animals. We demonstrate the flexibility of SAMPL’s acquisition suite using three common model organisms. By changing SAMPL’s thresholds (Table S2), we could acquire data from three different organisms: *Drosophila melanogaster* climbing behavior (Figures 2A and 2B), continuous locomotion in *Caenorhabditis elegans* (Figures 2C and 2D), and swimming in *Danio rerio* (Figures 2E and 2F). We present raw video from the epochs in Figure 2 together with plots of real-time image processing (fly and worms in Video S2 and fish in Video S3). These results demonstrate SAMPL’s excellent flexibility and robustness in real-time recording and analysis of vertical locomotion of small animals.

### SAMPL validation: Measuring postural and locomotor kinematics in real time

Next, to demonstrate how SAMPL facilitates efficient collection of high-quality kinematic data, we gathered a dataset from larval zebrafish (7–9 days post-fertilization [dpf]) that swam freely in the dark. A typical experimental repeat consisted of two sequential 24 h sessions using 3 SAMPL boxes. Data were pooled across 27 repeats for subsequent analysis of kinematics. Each 24 h behavior session yielded on average 1,223 ± 481 bouts per day for the standard chamber (6–8 fish) and 1,251 ± 518 bouts per day for the narrow chamber (1–3 fish). While not analyzed, running a single fish in the narrow chamber yielded 891 ± 903 bouts over 24 h. Based on the number of apparatuses used, we estimate that a similar dataset (total n = 121,979 bouts) could be collected in 2 weeks using a single SAMPL rack.

We first used our data to establish basic distributions of locomotion and posture. We used SAMPL’s processing algorithm to extract the following information in real-time: (1) pitch, defined as the angle between the long axis of the fish’s body and the horizon (Figure 2E) and (2) x (azimuth) and z (elevation) coordinates of the center of the pixels that correspond to the fish. After collection, we used SAMPL’s processing suite to extract basic postural kinematics during swimming. Zebrafish larvae swim in discrete periods of translation called “swim bouts” (Figure 2F).^16,20^ We defined swim bouts as periods where the instantaneous speed exceeds 5 mm/s (Figure 2F, dashed line). The time of the peak speed was defined as t = 0 ms (Figure 2F, cyan lines). Swim bouts were aligned to peak speed for extraction of kinematic parameters; the periods 250 ms before and 200 ms after peak speed were reserved for future analysis. We observed that zebrafish larvae swim predominantly at slower speeds, with mean and standard deviation measured 12.90 ± 4.91 mm/s, on par with previous reports.^16,20–22^ Larvae showed a broad distribution of postures evaluated at peak speed (8.48° ± 15.23°) with a positive (nose-up) average, suggesting that SAMPL detected a variation of nose-up and nose-down swim bouts. SAMPL can thus rapidly acquire a rich dataset of spontaneous locomotor behavior and a wide range of “natural” postures.

### SAMPL validation: Extracting key parameters of balance and vertical navigation in zebrafish

**Figure 3.**
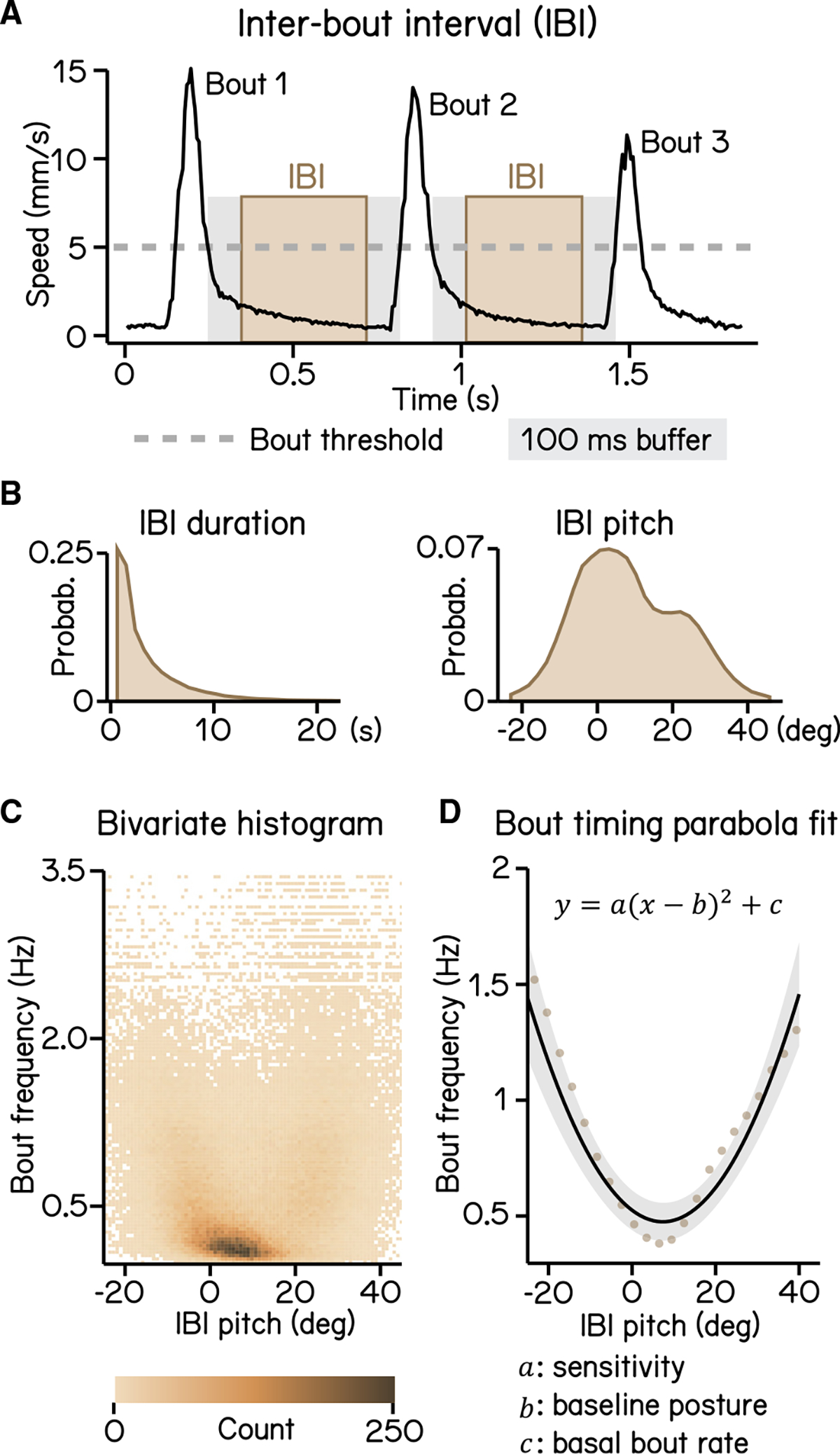
Modeling timing of swim bouts reveals larval sensitivity to pitch changes (A) An inter-bout interval (IBI; brown area) is defined as the duration when swim speed is below the 5 mm/s homeostasis threshold (dashed line) between two consecutive bouts with a 100 ms buffer window (gray area) deducted from each end. (B) Distribution of IBI duration (left) and mean pitch angle during IBI (right). (C) Bivariate histogram of bout frequency and IBI pitch. Bout frequency is the reciprocal of IBI duration. (D) Bout frequency plotted as a function of IBI pitch and modeled with a parabola (black line, R ^2^ = 0.14, mean ± SD). Brown dots indicate binned average of IBI pitch and bout frequencies calculated by sorting IBI pitch into 3°-wide bins. For all panels, n = 109,593 IBIs from 537 fish over 27 repeats. See also Table S3.

**Figure 4.**
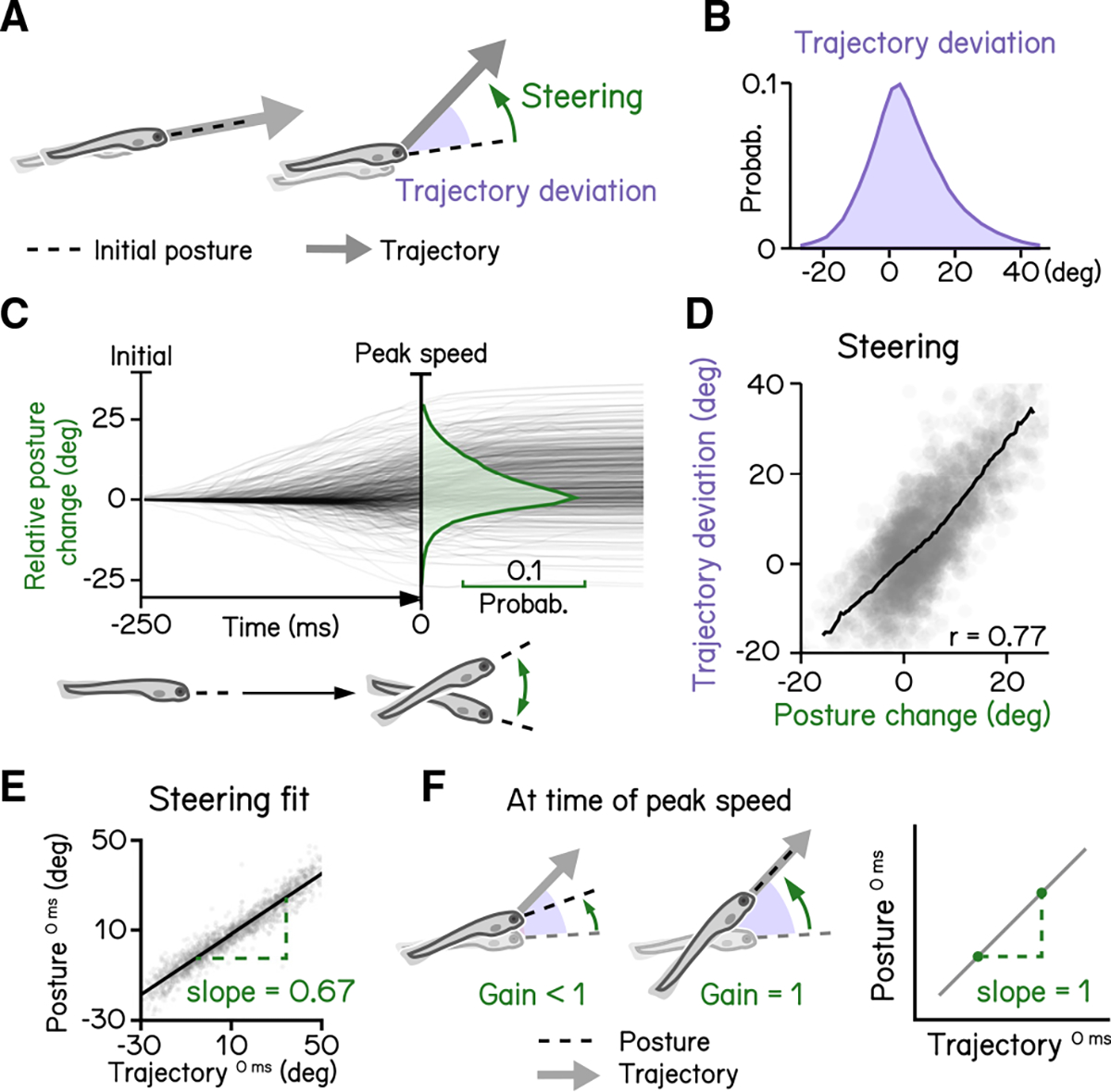
Larval vertical navigation is led by steering toward trajectory (A) Schematic illustration of two climbing mechanics: (1) a larva may generate a thrust (arrow) toward the pointing direction (dashed line) at the initial of a bout (left), and (2) a larva can steer (green arrow) toward an eccentric angle before the thrust (right). The offset between trust angle and the direction the larva point toward at bout initial is termed trajectory deviation (purple). (B) Distribution of trajectory deviation. (C) Changes of pitch angles relative to initial pitch plotted as a function of time(dark lines) overlaid with distribution of pitch change at time of peak speed (green). (D) Trajectory deviation (purple) plotted as a function of posture changes from bout initial to time of the peak speed (green). Black line indicates binned average values. Positive correlation between trajectory deviation and posture change demonstrates that larvae steer toward the trajectory of the bout. (E) To measure the gain of steering compared with trajectory deviation, pitch angles at time of the peak speed are plotted as a function of trajectory. Steering gain is determined as the slope of the fitted line (Pearson’s r = 0.96). (F) Schematic illustrations demonstrating how steering gain associates steering (green arrows) with trajectory deviation (purple). For all panels, n = 121,979 bouts from 537 fish over 27 repeats. See also Table S3.

**Figure 5.**
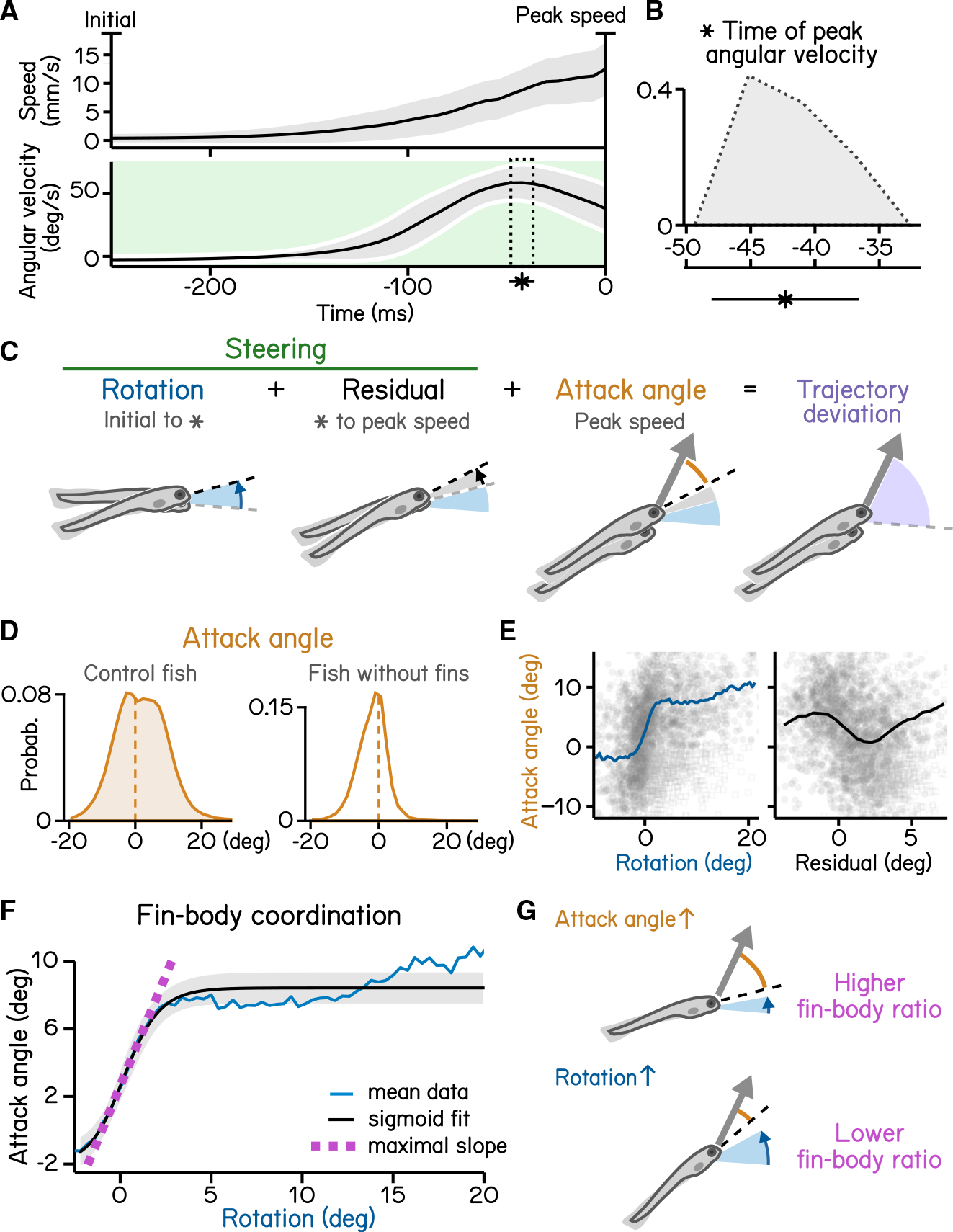
Steering requires coordination of fin and body (A) Swim speed (top) and angular velocity (bottom) plotted as a function of time. Angular velocity peaks (asterisk and dotted area, mean ± SD) during steering phase (green) before time of the peak speed. Angular velocity is adjusted by flipping signs of bouts with nose-down rotations during steering (mean ± SD across experimental repeats). Shaded region in the top panel indicates mean ± SD across all quantified swim bouts. (B) Histogram of time of peak angular velocity, binned by frame, across experimental repeats with mean ± SD plotted below. (C) Illustration of components that contribute to trajectory deviation. Larvae rotate their bodies starting from bout initial (blue) and reach peak angular velocity (asterisk) before peak speed. Any rotation generated during decrease of angular velocity is considered residual (gray). At time of peak speed, there is an offset between the pitch angle (dashed line) and bout trajectory (arrow), which is termed the attack angle (orange). Body rotations, residual, and attack angle add up to trajectory deviation. (D) Distribution of attack angles in control fish(left) and fish after fin amputation (right). Dashed lines indicate 0 attack angle. (E) Attack angles plotted as a function of body rotations (left, blue) or residual rotations (right). Rotations and residuals are sorted into 0.5°-wide bins for calculation of binned average attack angles. Swim bouts with negative attack angles while having steering rotations greater the 50th percentile (hollow squares) were excluded for binned-average calculation. (F) Attack angles plotted as a function of body rotations (blue line) and fitted with a logistic model (black line, R^2^ = 0.31, mean ± SD). Fin-body ratio is determined by the slope of the maximal slope of the fitted sigmoid (magenta). Rotations are sorted into 0.8°-wide bins for calculation of binned average rotations and attack angles (blue line). Swim bouts with negative attack angles while having steering rotations greater the 50th percentile were excluded for sigmoid modeling. (G) Schematic illustration of how fin-body ratio reflect climbing mechanics. For all panels, n = 121,979 bouts from 537 fish over 27 repeats. See also Table S3.

**Figure 6.**
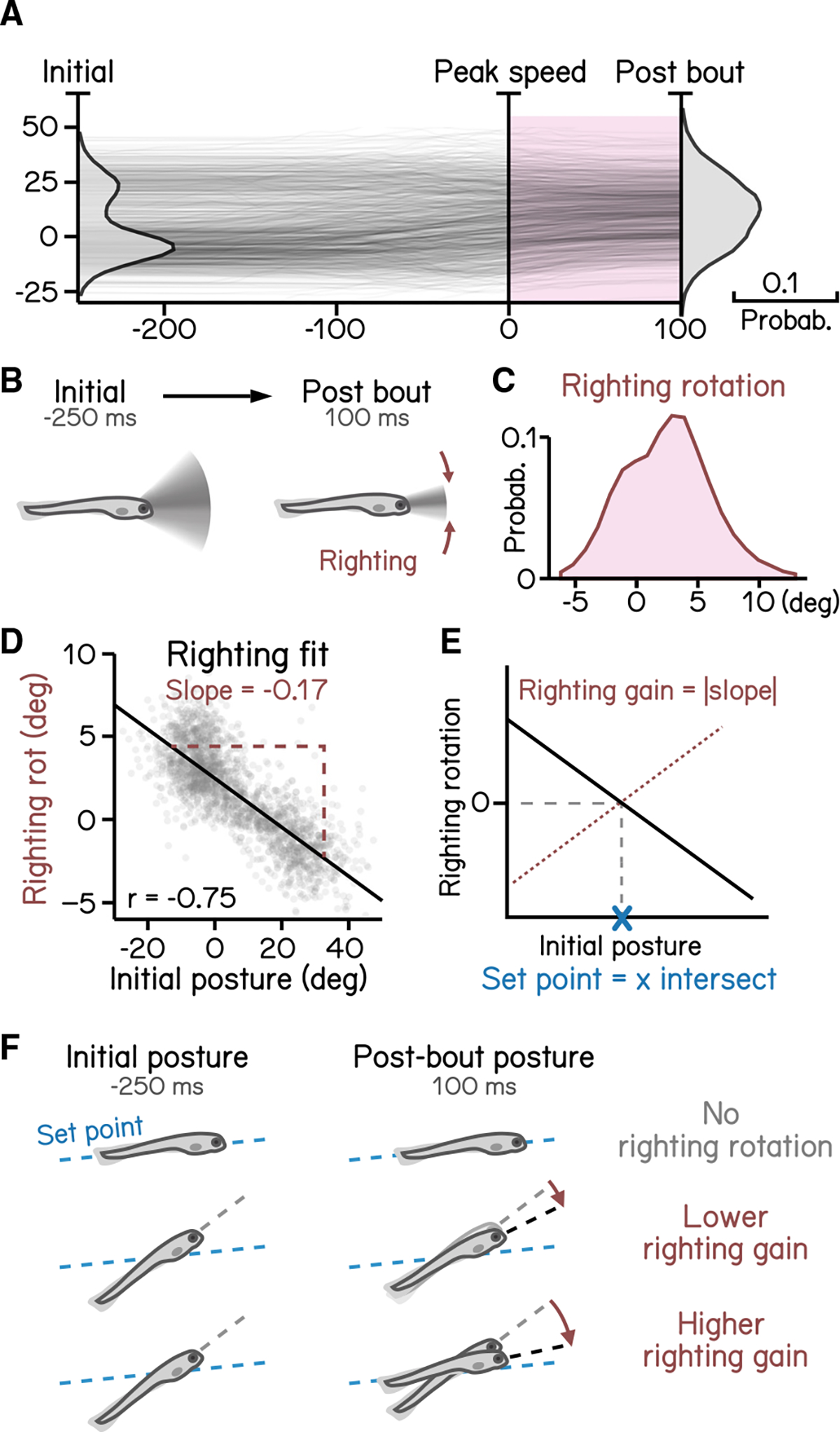
Righting rotation restores posture after peak speed (A) Pitch angles plotted as a function of time (dark lines) overlaid with distribution of pitch angles before (left) and after bouts (right). Red area indicates duration after peak speed when pitch distribution narrowed. (B) Illustration of righting behavior. Larvae rotate (red arrows) toward more neutral posture after peak speed. (C) Distribution of rotation during righting (red in A). (D) Righting rotation plotted as a function of initial pitch angles. (E) Righting gain is determined by the absolute value of the slope (red dotted line) of best fitted line (black line). The x intersect of the fitted line determines the set point (blue cross) indicating the posture at which no righting rotation results. (F) Schematic illustration of righting rotation (red arrows), righting gain, and setpoint (blue dashed line). For all panels, n = 121,979 bouts from 537 fish over 27 repeats. See also Table S3.

SAMPL includes data analysis and visualization code (Python source and sample datasets provided) optimized to extract key parameters of balance and locomotion from larval zebrafish. We use our “2 week” dataset to demonstrate that SAMPL can resolve these four parameters: control of movement timing^16^ (Figure 3), control of steering to climb/dive^17^ (Figure 4), coordination between trunk and fin^18^ (Figure 5), and control of posture-stabilizing rotations^17^ (Figure 6).

We conclude that SAMPL’s resolution and throughput allow rapid and deep insight into each parameter, as detailed below. Data analysis using the provided scripts on the provided dataset runs in 30 min on a typical analysis computer (M1 processor, 16 GB RAM). Full details of analysis/visualization are provided in Note S4, and a step-by-step guide to set up the relevant environment and to run experiments is provided in Note S5.

Proper balance requires active stabilization. Zebrafish larvae are front heavy and therefore subject to destabilizing torques in the pitch (nose-up/nose-down) axis. Swim bouts counteract the resultant forces, stabilizing the fish. Zebrafish larvae learn to initiate swim bouts when unstable.^16^ We first defined movement rate as the reciprocal of the inter-bout interval (Figures 3A and 3B). More extreme postures were associated with higher movement rate (Figure 3C), with a parabolic relationship (Figure 3D; R^2^ = 0.14). We expect that the majority of the residual variance reflects a previously reported dependence of movement timing on angular velocity.^16^ The three coefficients of the parabola represent the baseline posture, the basal rate of movement, and—key to our analysis—the degree to which postural eccentricity relates to movement rate, or “sensitivity” (Figure 3D). SAMPL therefore permits efficient quantification of a crucial posture-stabilizing behavior: the relationship between perceived instability and corrective behavior.

Like most animals, larval zebrafish go where their head points. To adjust their vertical trajectory (i.e., to climb or dive), larvae must rotate their bodies away from their initial posture, pointing in the direction they will travel (Figures 4A and 4B).^17,23^ Previous work^17^ established that steering rotation in larvae swimming spontaneously occurs mostly before they reach the peak speed (Figure 4C). A larva’s steering ability reflects the relationship between the change in posture before the peak speed and the resultant deviation in trajectory (Figure 4D). We parameterized steering as the slope (gain) of the best-fit line between posture and trajectory evaluated at the time of peak speed (Figure 4E). A gain of 1 indicates that the observed trajectory could be explained entirely by the posture at the time of peak speed (Figure 4F). SAMPL revealed that 7 dpf larvae exhibit an average steering gain at 0.67, suggesting an offset between posture and trajectory at the time of peak speed (Figure 4E; R^2^ = 0.92). SAMPL allows us to infer how effectively larvae steer using axial (trunk) musculature to navigate the water column.

To climb (Figures 5A and 5B), fish generate lift with their pectoral fins, assisting steering rotations and subsequent axial undulation.^24,25^ Larval zebrafish learn to climb efficiently by coordinating their trunk and fins.^18^ We defined the attack angle, or the additional lift associated with each climb, as the difference between the steering-related changes and the resulting trajectory (Figure 5C). We evaluated attack angle after pectoral fin loss, revealing a clear contribution to climbs (Figure 5D). Next, we demonstrate a positive correlation (with rectification and asymptote) between steering-related rotations and fin-based attack angle (Figure 5E, left). Notably, after peak angular velocity, rotations are poorly correlated with attack angles (r = −0.17) (Figure 5E, right). These residuals reflect the initial angular deceleration as fish reach their peak speed (Figure 5A). We parameterize the relationship between the initial rotation and the attack angle using logistic regression (Figure 5F; R^2^ = 0.31). The regression reveals the maximal slope of the sigmoid relating steering and lift (Figure 5G). We named this slope “fin-body ratio,” as it describes how larvae distribute labor between axial and appendicular muscles, i.e., between trunk (steering) and fins (lift), as shown in previous work.^18^ SAMPL thus permits efficient inference of coordinated behavior.

Larvae must actively maintain their preferred posture in the pitch axis. To do so, they rotate partially toward their preferred orientation as they decelerate (Figures 6A–6C). The magnitude of these rotations scales with the eccentricity of their posture before a swim bout.^17^ We estimated the slope (−0.17) of the line that related initial posture and the amount the fish that rotated back toward the horizontal (Figure 6D; R^2^ = 0.56). As the behavior is corrective, the relationship is negative; we therefore define the gain of righting as the inverse of the slope (Figure 6E). We further define the “set point” as the point where an initial posture would be expected to produce a righting rotation of zero (Figures 6E and 6F). SAMPL facilitates quantification of corrective reflex abilities (gain) and associated internal variables (set point).

Taken together, our estimates of key posture and locomotor parameters establish that SAMPL can rapidly generate datasets that permit rich insight into the mechanisms of balance and vertical navigation.

### SAMPL can resolve slight variations in posture control strategies across genetic backgrounds

To be useful, SAMPL must resolve small but systematic differences in key measures of posture and vertical locomotion. Even among isogenic animals reared in controlled environments, genetic differences contribute to behavioral variability.^26–33^ The “2 week” dataset analyzed in Figures 3, 4, 5, and 6 included data from three different genetic backgrounds. Larvae for experiments were generated by crossing the same clutch of wild-type adults (mixed background) to zebrafish of three different strains: AB (n = 62,457 bouts, N = 225 fish over 10 experimental repeats), Sanger AB Tübingen (SAT) (n = 27,990 bouts, N = 117 fish over 7 experimental repeats), and the lab wild type (n = 31,532 bouts, N = 195 fish over 10 experimental repeats), which resembles real-world approaches where a key transgenic line is often crossed to different backgrounds for experiments. To capture the full variance in the dataset, we took a conservative approach by calculating kinematic parameters for individual experimental repeats (n = 4,518 ± 1,658 bouts). We assayed SAMPL’s sensitivity by asking (1) if there were detectable differences in the four parameters defined in Figures 3, 4, 5, and 6 and (2) if these differences were systematic.

Qualitatively, larval zebrafish of the same age swim similarly; as expected, the magnitude of change across strains that we observed in Figure S2 is quite small. Nonetheless, SAMPL could resolve systematic variations in locomotion behavior and balance abilities among larvae of different strains (Figure S2). AB larvae exhibited the best posture stability, demonstrated by the lowest standard deviation of inter-bout interval (IBI) pitch compared with the other two strains (Figure S2C). Correspondingly, AB larvae had the highest bout frequency (Figure S2B), sensitivity to posture changes (Figure S2E), and righting gain (Figure S2K), all of which contribute positively to their higher posture stability. These results demonstrate that SAMPL is capable of detecting inter-strain variations in locomotion and balance behavior.

In contrast, larvae of different ages adopt different strategies to stabilize posture and navigate in depth.^16–18^ To contextualize the magnitude of strain-related differences, we gathered a longitudinal dataset by measuring behavior from the same siblings of the AB genotype at three timepoints: 4–6, 7–9, and 14–16 dpf (Table S3). We observed that the standard deviations of IBI pitch for 4 and 14 dpf larvae were 38.1% higher and 11.3% lower, respectively, than the average result of 7 dpf larvae (Table S3). Across strains at 7 dpf, the variation was much smaller: from 11.8% higher to 11.2% lower. Similarly, relative to 7 dpf larvae, the sensitivity of 4 dpf larvae was considerably lower (42.5%) and increased to 23.6% higher by 14 dpf (Table S4); variations among 7 dpf strains were up to 10% lower and 15.4% higher.

Our data support three key conclusions. First, SAMPL can uncover small, systematic differences in the way fish swim and stabilize posture. Second, SAMPL can make longitudinal measures of the same complement of animals as they develop. Third, relative to development, the effect of genetic background is small. We conclude that SAMPL’s capacity to resolve small differences supports its usefulness as a tool screen for modifiers of postural control and vertical locomotor strategies.

### Estimating SAMPL’s resolution

Our dataset establishes SAMPL’s ability to resolve small kinematic differences between cohorts. How does SAMPL’s power change as a function of the size of the dataset? We used resampling statistics to estimate SAMPL’s resolution as a function of the number of the bouts (STAR Methods). To ensure our most conservative estimate, we resampled data combined across AB, SAT, and wild-type (WT) genotypes at 7 dpf.

**Figure 7.**
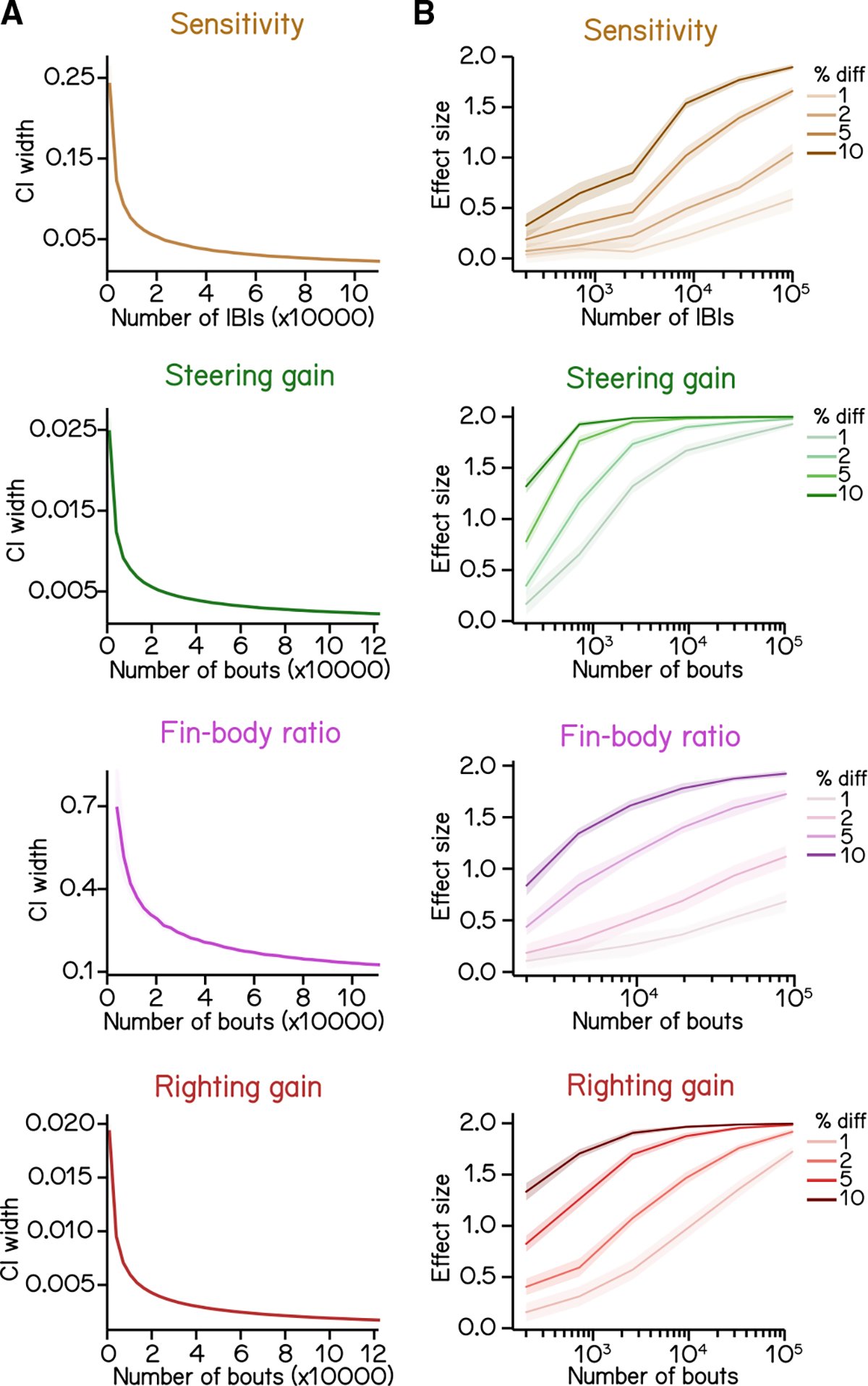
Statistics of regression analysis for swim kinematics (A) Confidence interval (CI) width of kinematic parameters plotted as a function of sample size at 0.95 significance level (mean ± SD as ribbon). Errors were estimated by resampling with replacement from the complete dataset. (B) Effect size plotted as a function of sample size at various percentage differences. Mean ± SD. Refer to STAR Methods for details of computation.

As expected, the width of the confidence interval for any estimated parameter decreased with the number of bouts (Figure 7A). The most challenging parameter to estimate is coordination between fin and trunk (fin-body ratio) The steepness with which the confidence interval width decreases follows the number of regression coefficients necessary for each measure: fin-body ratio (4 parameters), bout timing (3 parameters), and steering or righting (2 parameters). We therefore propose that these particular measures can serve as a general guide for the challenge of estimating parameters within a SAMPL dataset.

A fundamental challenge for all screens is determining the sample size required to correctly reject the null hypothesis.^34^ We address this question by asking how much data one would need to gather in order to detect meaningful effects. We simulated the difference of particular magnitudes by imposing an offset on each parameter (sensitivity, steering gain, fin-body ratio, and righting gain) while preserving the original variance (STAR Methods). Offsets were expressed as a fractional difference, and resampling was used to estimate the effect size one would see as a function of the number of bouts/IBIs when comparing kinematic parameters between the original dataset and the dataset with an imposed effect (STAR Methods).

Broadly, we find that for all kinematic parameters, the smaller the percentage change, the larger the required sample size (Figure 7B). Steering and righting gains require the fewest bouts to detect a 1%–2% change with an effect size >0.5 (Figure 7B, green and red). However, sensitivity and fin-body ratio require relatively larger datasets to confidently discriminate small changes (Figure 7B, brown and magenta). We conclude that the full “2 week” dataset we generated using SAMPL (n = 121,979 bouts) is sufficient to reveal any biologically relevant differences between two conditions.

In summary, these simulations demonstrate that a single SAMPL rack divided into two conditions (6 apparatuses/each) could, in two standard 48 h runs, generate sufficient data to resolve meaningful differences in postural and locomotor kinematics between two conditions. We provide detailed instructions in Note S5 addressing experimental design strategies to maximize SAMPL’s resolution.

## DISCUSSION

We present SAMPL, a scalable solution to measure posture and locomotion in small, freely moving animals. We start with a brief overview of the hardware and software, with comprehensive guides for every aspect of SAMPL’s hardware and software included in the appendices. Next, we illustrate SAMPL’s flexibility with raw video and real-time measurements from three common model organisms: *Drosophila melanogaster* (fly), *Caenorhabtitis elegans* (worms), and *Danio rerio* (zebrafish). To illustrate the depth of insight accessible using SAMPL, we explored a dataset—consisting of 2 weeks’ worth of data—that illuminates four key parameters of zebrafish navigation in depth: bout timing, steering, fin-body coordination, and righting. We made two discoveries using SAMPL’s analysis suite: (1) systematic changes to zebrafish posture and locomotion across genetic backgrounds and (2) that these changes were small relative to variation across developmental time. Finally, we use our dataset to define SAMPL’s resolution: how much data an experimenter would need to collect to detect meaningful effects. Taken together, SAMPL provides a screen-friendly solution to investigate vertical locomotion and/or other behaviors using common small-model organisms and a turnkey solution to study balance in larval zebrafish. More broadly, our approach serves as a template for laboratories looking to develop or scale their own hardware/software. Below, we detail SAMPL’s innovations and limitations and make a case for screens to address unmet clinical needs for balance disorders.

### SAMPL’s innovations

One of SAMPL’s key innovations is to measure vertical behavior, where the effects of gravity play a role. The overwhelming majority of studies monitor animal behavior from above, where animals are constrained to a horizontal plane. For most animals—especially those that swim or fly—vertical navigation and its neuronal representation^35,36^ is vital. Further, maintaining posture in the face of gravity is a universal challenge,^37–39^ particularly as animals develop.^16,40^ SAMPL can illuminate animal trajectories during exploration of depth.

SAMPL reduces the dimensionality of behavior along a number of axes in real time. First, by focusing on a homogeneous part of the behavioral arena, SAMPL bypasses a number of imaging challenges and difficulties involved in interpreting behavior along arena walls.^41^ Second, by rejecting frames with multiple animals in view at the same time, SAMPL incorporates animal-to-animal variability^4^ within each estimated parameter without having to keep track of individuals; the narrow chamber (Figure 1E) is ideal for single-animal experiments if such variability is of interest. Third, while large enough to permit unconstrained behavior, the anisotropic dimensions of SAMPL’s behavioral arenas (Figure 1E) facilitate measurements in the vertical axis. SAMPL’s design choices thus facilitate rapid extraction of behavioral parameters relevant for posture and locomotion.

SAMPL was designed to scale efficiently. Data are gathered by a compiled executable, allowing SAMPL to run three apparatus off a single computer, reducing costs and space. A SAMPL rack consists of 12 apparatus running off four computers with a footprint of 24″ × 36” × 81.5” (L × W × H). The key components such as the camera are readily available from multiple suppliers. Taken together, SAMPL can be used immediately to screen and/or to provide videographic data from freely moving animals at scale.

Our dataset, gathered in 2 weeks, illustrates the power of SAMPL’s analysis/visualization workflow for studies of larval zebrafish balance. While SAMPL can and does save video, by design it extracts only three parameters in time: the (x,z) coordinates of the animal and the angle between the body and the horizon. As we demonstrate here, this small set of parameters defines the behaviors larval zebrafish use to swim and balance in depth: bout timing (Figure 3), steering (Figure 4), fin-body coordination (Figure 5), and righting (Figure 6). While each parameter has been previously defined,^16–18^ the data we present here illustrate differences across genetic backgrounds and development and allow granular estimation of statistical sensitivity. Taken together, SAMPL’s focus facilitates exploration of unconstrained vertical behavior.

### Comparisons with other approaches

Here, we discuss SAMPL’s advantages by comparing it with other available tools for measuring *Drosophila, Caenorhabtitis elegans*, and zebrafish behavior.

### SAMPL for measuring *Drosophila* behavior

SAMPL offers advantages over previous methods for measuring negative gravitaxis, an innate behavior of *Drosophila melanogaster*.^42^ The most widespread method, called the bang test, consists of banging flies down inside a vertical tube and then counting the number of flies that walk an arbitrary vertical distance in an arbitrary amount of time.^42–45^ This method startles the flies, which may confound the behavior, and the flies are limited in directional choice. Using SAMPL, a measurement of fly vertical position and orientation is instantaneously acquired without needing to startle the flies. Another *Drosophila* gravitaxis assay is the geotaxis maze,^46^ which allows the flies to make a series of up or down choices as they move across the maze toward a light. While the flies are not startled in this assay, they are still constrained to moving only up or down. SAMPL’s high-resolution camera permits continuous monitoring of free vertical walking behavior, as well as high-resolution monitoring of head, wing, leg, and antenna positions. While SAMPL has been designed to monitor behavior in the vertical plane, the hardware and software strategies we have developed for high-throughput recording could be similarly adapted to increase the throughput of measuring other *Drosophila* behaviors such as grooming,^47^ sleep,^48^ courtship,^49^ and aggression.^50^ Because SAMPL has both high-resolution recording and the ability to scale, screening through microbehaviors like head tilting or limb positioning is possible. Notably, an earlier version of SAMPL’s detection algorithm was successfully used for data acquisition in a fly olfactory behavior assay^51,52^ with minimal changes. Taken together, SAMPL’s resolution, throughput, and adaptability complement and extend current approaches to measure *Drosophila* behavior, particularly in the vertical axis.

### SAMPL for measuring *Caenorhabtitis elegans* behavior

The simple nervous system of *Caenorhabtitis elegans* is an ideal model to study neural circuits that control posture and movement. *Caenorhabtitis elegans* possess a rich and tractable repertoire of motor control.^53^ For example, a pattern generator creates sinusoidal waves of muscle contraction that propel *Caenorhabtitis elegans* on a solid substrate, and these sinusoidal movements are sculpted by proprioceptive feedback.^54^ Proprioceptive feedback also controls transitions between sinusoidal crawling and non-sinusoidal bending that can propel animals in a liquid environment.^55–57^ Other sensory stimuli elicit coordinated motor responses that are critical for navigation. Decreasing concentrations of attractive odorants and gustants triggers reversals followed by a pirouette or omega bend, which results in a large-angle turn that reorients animals.^58,59^ A distinct navigation behavior involves precise steering of an animal as it follows an isotherm in a temperature gradient^60,61^ or tracks a preferred concentration of gustant.^62^ The resolution and scalability of SAMPL offers the opportunity to determine the cellular, molecular, and genetic underpinnings of these diverse motor control mechanisms.

*Caenorhabtitis elegans* behavior becomes complex in enriched 3D environments, with animals using strategies for exploration and dispersal not seen under standard laboratory conditions.^63^ Behavior trackers that have been used to study *Caenorhabtitis elegans* kinematics are generally restricted to analysis of behaviors on a surface. By contrast, SAMPL measures behavior in a volume and is well suited to the study of newly discovered behaviors that are only expressed in environments that vary across depth. One such example is gravitaxis, where *Caenorhabtitis elegans* display both positive^64^ and negative gravitaxis,^65^ underscoring the need for additional pipelines to test behavior.^66^ The data we present here establish that SAMPL offers a complement to existing pipelines for *Caenorhabtitis elegans* assays of behavior in the vertical dimension.

### SAMPL for measuring zebrafish behavior

SAMPL joins a decades-long tradition of apparatus that has, collectively, established the larval zebrafish as a key vertebrate model to understand the neural control of posture and locomotion.^13–15^ Broadly, these devices sit on a continuum that represents a trade-off between imaging resolution and throughput. At one end, exquisite measures of tail or eye kinematics are available when imaging single animals that are partially restrained^67^ or contained in a small field of view.^68^ Such devices are particularly useful when combined with imaging or perturbations of neuronal activity but at the cost of throughput. At the other end are devices that measure activity when single animals are constrained to small arenas, such as the ~8 mm^2^ wells in a 96-well plate.^6,69–71^ These devices lend themselves well to screens and offer commercial options, but the range of behaviors is compressed.^72^ Like other attempts to preserve high-resolution kinematic information while accommodating natural unconstrained behavior,^22,73–78^ SAMPL sits between these two extremes, joining other open-source software packages such as Stytra^79^ and Zebrazoom.^80^ We see SAMPL as a complementary tool. SAMPL’s emphasis on vertical behavior and its scalability position it to leverage the advantages of the zebrafish model for screens—either as a primary resource or to follow up on promising “hits” identified with higher-throughput approaches.^6^

### Screening

Balance disorders present a profound and largely unmet clinical challenge.^19^ Because the neuronal architecture for balance is highly conserved and the fundamental physics (i.e. gravity is destabilizing) is universal, animal models represent a promising avenue for discovery. Due to their size, low cost, molecular accessibility, high fecundity, and conserved biology small animals—both vertebrates and invertebrates^81^—have long been used in successful screens of both candidate genes,^82^ peptides,^83^ and therapeutics.^84,85^ Zebrafish are an excellent exemplar, particularly in the space of neurological disorders,^3^ with well-established approaches for candidate gene screens,^2,5^ peptides,^86^ small molecules,^87–91^ and disease models.^92^ Using SAMPL with zebrafish, our dataset establishes a foundation to screen for balance modifiers in health and disease.

One particular arena where zebrafish screens for balance/posture could have a profound impact is in addressing the unmet therapeutic need that exists for a neurodegenerative tauopathy: progressive supranuclear palsy (PSP). PSP is initially characterized by balance impairments, falls, vertical gaze palsy, and rigidity.^93,94^ Falls are central to early^95^ PSP presentation and diagnosis^96,97^ and lead to fractures and hospitalization.^96,98^ Currently, no treatments improve balance. Studies of posture,^99–103^ graviception,^104^ reflexes,^105–108^ electromyography,^109,110^ and neural balance circuits in PSP^103,111–115^ are often underpowered and inconsistent and have yet to identify the specific mechanism or substrate causing falls. Like most genes and subcortical structures^116–123^ the genetic and anatomical substrates of PSP are conserved between humans and zebrafish.^124–127^ Here, using SAMPL, we define behavioral endpoints that reflect how pathological zebrafish might “fall.” By establishing SAMPL’s resolution, our data lay the foundation for impactful discovery in the space of a neurodegenerative disorder with balance pathology.

### Future prospects

SAMPL uses low-cost videographic and computing hardware to make behavioral measurements. By optimizing scalability, resolution, and extensibility, SAMPL allows experimenters to rapidly measure unconstrained behavior as animals navigate in depth. We have used SAMPL with a model vertebrate, zebrafish, to gain insight into posture and vertical locomotion and to lay the groundwork for future screens. A wide variety of neurological disorders present with balance and locomotor symptoms. SAMPL offers a way to both understand the fundamental biology of balance as well as means to evaluate candidate therapeutics to address this unmet need. More broadly, SAMPL stands as an exemplar and resource for laboratories looking to develop, adapt, or scale videographic apparatus to measure behavior in small animals.

### Limitations of the study

Any apparatus necessarily reflects a set of trade-offs. Consequentially, each of SAMPL’s innovations can reasonably be recast as a limitation depending on experimental priorities. For example, SAMPL’s focus on a subset of space and parameters is ill suited to reconstruct a catalog of behaviors from videographic measurements, i.e., a computational ethogram.^11,20^ Similarly, SAMPL assumes that the animal’s trajectory reflects coordinated use of its effectors (limbs/trunk/wings). While SAMPL’s videos would be an excellent starting point for marker-less pose estimation, detailing the links between effector kinematics and resultant changes to posture and trajectory may be better served by a multicamera setup.^8,9^ SAMPL’s processing is exclusive to one animal; other approaches are therefore necessary to resolve social interactions.^7,128^ Finally, SAMPL’s analysis/visualization toolset incorporates priors for movement of zebrafish only—studies of other species would require a moderate investment of effort.

## STAR★METHODS

### RESOURCE AVAILABILITY

#### Lead contact

Further information and requests for resources and reagents should be directed to and will be fulfilled by the lead contact, Dr. David Schoppik (schoppik@gmail.com).

#### Materials availability

This study did not generate new unique reagents.

#### Data and code availability

- Behavior data have been deposited at the Open Science foundation and are publicly available as of the date of publication. DOIs are listed in the key resources table.
- All original code has been deposited at the Open Science foundation and is publicly available as of the date of publication. DOIs are listed in the key resources table.
- Any additional information required to reanalyze the data reported in this paper is available from the lead contact upon request.

### EXPERIMENTAL MODEL AND SUBJECT DETAILS

All procedures involving larval zebrafish (*Danio rerio*) were approved by the New York University Langone Health Institutional Animal Care & Use Committee (IACUC). Zebrafish larvae were raised at 28.5°C on a standard 14/10 h light/dark cycle at a density of 20–50 larvae in 25–40 mL of E3 medium before 5 days post-fertilization (dpf). Subsequently, larvae were maintained at densities under 20 larvae per 10 cm Petri dish and were fed cultured rotifers (Reed Mariculture) daily. Larvae that had their behavior measured at 14 dpf were raised as stated above before being moved to 2 L tanks with 300 mL of cultured rotifers at 9 dpf. At 13 dpf, they were transferred back to petri dishes with E3 medium for adaptation.

Larvae with different strains were achieved by crossing Schoppik lab strain with a mixed AB, TU, and WIK background to three different wild-type strains: AB (Zebrafish International Resource Center), mixed background of AB/WIK/TU, or SAT (Zebrafish International Resource Center). Reference parameter values in Table S3 for 4, 7, 14 dpf fish were gathered using the AB strain fish.

*Drosophila melanogaster* (*w*^1118^) were raised at 23° on standard cornmeal-agar food under a 12/12 light/dark cycle.

*Caenorhabditis elegans* (*C. elegans*) were grown at 20° on nematode growth medium agar plates seeded with *Escherichia coli* OP50.^139^

### METHOD DETAILS

#### Behavior experiment

Larvae at desired age (4, 7, or 14 dpf) were transferred from petri dishes to behavior chambers at densities of 5–8 per standard chamber and 2–3 per narrow chamber with 25–30/10–15 mL of E3, respectively. After 24 h, behavior recording was paused for 30–60 min for feeding where 1–2 mL of rotifer culture was added to each chamber. Larvae were removed from the apparatus 48 h after the start of the recording.

Behavior measurement in this manuscript were collected from 27 clutches of zebrafish larvae between 7 and 9 dpf under constant darkness. 4 dpf and 14 dpf reference parameter values in Table S3 were collected from 10 clutches of zebrafish larvae under constant darkness. Finless data was generated using 4 clutches of larvae under constant darkness. For all experiments, a single clutch of larvae produces one experimental repeat with at least 3 behavior boxes each containing 5–8 larvae per standard chamber or 2–3 fish per narrow chamber.

For *Drosophila* recording, four flies were transfered to a narrow chamber. A small piece of water-dampened kimwipe was put at the bottom of the chamber to maintain humidity. An n acrylic plug was secured at the top to prevent them from escaping the chamber. We secured the chamber with the flies in the SAMPL apparatus and performed the standard SAMPL experiment using recording parameters provided in Table S2.

To image swimming *C. elegans*, eight starved N2 adult hermaphrodites were transferred to a narrow chamber filled with 15 mL M9 buffer (3 g/L KH_2_PO_4_; 6 g/L Na_2_HPO_4_; 0.5 g/L NaCl; 1 g/L NH_4_Cl) which was secured in the SAMPL apparatus. Behavior recording was started immediately afterward. Refer to Table S2 for SAMPL thresholds for *C. elegans* detection.

#### Fin amputation

6 dpf zebrafish larvae were anesthetized in 0.02% tricaine methanesulfonate (Syndel) and transferred to 3% Methylcellulose (Sigma). Fin amputation was done by removing pectoral fins using fine forceps (FST). Specifically, one pair of forceps was used to stabilize the head of the fish and a second pair was used to grab the joint and pull off the fins. Finless larvae were washed three-times in E3 and fed with cultured rotifers before behavior assessment at 7 dpf.

#### Video acquisition

Video S1 was captured using Sigma fp digital camera (Sigma Co.). Video footage was edited and annotated using Premiere Pro (Adobe). Videos S2 and S3 was captured with the innate video capture function in SAMPL software using recording parameters described in Table S2. Video S3 was edited using Adobe Premiere Pro (Adobe) to combine with timeseries data.

### QUANTIFICATION AND STATISTICAL ANALYSIS

#### Behavior analysis

Behavior data was analyzed using the Python analysis pipeline SAMPL_analysis_visualization. SAMPL_analysis() function was used to calculate swim parameters, extract bouts and inter-bout intervals (IBIs) from the raw data, and align swim bouts by the time of the peak speed.

Each run of the experiment (recording from “start” to “stop”) generates one data file (*.dlm) containing recorded raw parameters including time stamp, fish body coordinates, fish head coordinates, pitch angle, epoch number and fish length at every time point. An epoch is defined by a duration where the number of detected pixels falls within the lower and upper threshold for recording, indicating detection of fish in the field of view.

To extract bouts from the raw data, first, swim features, such as speed, distance, trajectory, angular velocity, etc., were calculated using basic parameters and time interval. Next, epochs that were longer than 2.5 s, contain maximum swim speed greater than 5 mm/s, and pass various quality-control filters were selected for bout extraction. Epochs containing multiple bouts were segmented and truncated so that each detected bout contains data from 500 ms before to 300 ms after the time of the peak speed. Then, bouts containing 800 ms of swim data were aligned by the time of the peak speed and saved for further analysis.

All further quantification was performed on data during zeitgeber day, namely the 14 h light time for fish raising under 14/10 h light/dark cycle.

To calculate IBIs, epochs with multiple bouts are selected and the duration of swim speed below the 5 mm/s threshold between two consecutive bouts is calculated. A 100 ms buffer window is then deducted from each end of the duration to account for errors of swim detection (Figure 3A). Pitch angles during each IBI were averaged to generate an IBI pitch (Figure 3B).

Definition of other bout parameters can be found in Table S3. All bout parameters (except for kinetic parameters explained in the next section) reported in the main text and Table S3 are mean values across swim bouts collected from multiple experimental repeats. One experimental repeat is defined as behavior data collected from one clutch of fish over 48 h using at least three boxes.

#### Computation of kinetic parameters

To calculate larvae sensitivity to pitch changes (Figure 3), we plotted bout frequency as a function of IBI pitch. The data was modeled using a quadratic polynomial regression (least squares) defined by function:

$$y=a(x-b{)}^{2}+c$$

where the coefficient of the quadratic term a indicates sensitivity and the y-intersect c represents baseline bout rate.

To calculate steering gain (Figure 4), we first computed bout trajectory defined by the tangential angle of instantaneous trajectory. Pitch angles at time of peak speed were then plotted as a function of bout trajectories and modeled with linear regression (least squares). The slope of the best fitted line was termed the “steering gain.”

Time of peak angular velocity in Figure 5 was computed using adjusted angular velocity. First, pitch angles for each bout were smoothed by a window of 11 frames and used for calculate angular velocity. Next, we flipped the signs of angular velocity for bouts that started with nose-down rotation so that all bouts started with positive angular velocity. To calculate time of peak angular velocity, we took the median angular velocity at every time point across all bouts from the same experimental repeat and found the time for the peak. Peak angular velocity times across all experimental repeats were then averaged to generate mean peak time.

For fin-body coordination analysis (Figure 5), we selected swim bout that are faster than or equal to 7 mm/s. Bouts with steering rotations (posture change from −250 ms to 0 ms) greater than the 50^th^ percentile while having a negative attack angle were further excluded from analysis. To calculate fin-body ratio, we plotted attack angles as a function of early rotation. Attack angle is defined as the difference between bout trajectory and pitch at time of peak speed. Body change related to steering were calculated by subtracting pitch angles at time of max angular velocity by initial pitch. Attack angle-rotation plot was then fitted with a logistic function defined by

$$y=a+\frac{h}{1+e^{-k(x+b)}}$$

where h is the height of the sigmoid. Fin-body ratio was defined by the maximal slope estimated using kh/4.

To calculate righting gain and set point (Figure 6), righting rotation, defined by the pitch changes from time of peak speed to 100 ms after peak speed, was plotted as a function of initial posture. Righting gain was determined by the absolute value of the slope of the best fitted line. The x intersect of the fitted line determines the set point (Figure 6E, blue cross) indicating posture at which results in no righting rotation.

#### Estimating effects of sample size on statistical modeling of bout kinetics

For statistical analysis of swim kinetics (??), the 7 dpf constant dark behavior dataset was sampled for 20 times at given sample number for calculation of swim kinetics and CI width. Specifically, sensitivity is determined by the coefficient of the quadratic term of the fitted bout-timing parabola as stated above. To plot estimated error as a function of the number of IBI, sets of data with N number of IBIs were sampled from the 7 dpf constant dark behavior dataset. However, different from the calculation of R^2^ above, the total dataset was sampled for 20 times for each desired number of IBIs (N). Regression analysis was performed on each set of sampled data to calculate sensitivity and its standard error. Estimated errors were used to calculate CI width at 0.95 significance level using normal distribution for each sampled dataset.

Similarly, steering gain and righting gain and their estimated errors were calculated from N number of bouts sampled from the original dataset. Estimated error was used to calculate CI width at 0.95 significance level for each sampled dataset. Sampling at each N was repeated for 20 times to generate error bars on the CI widths.

Fin-body ratio was calculated from N number of bouts sampled from the original dataset and repeated 20 times for each N. Because fin-body ratio is determined as the maximal slope of the sigmoid which is given by kh/4, the variance of fin-body ratio (slope) is calculated using formulation

$$V_{\mathrm{slope}}=\left(E_{k}^{2}\times V_{h}+E_{h}^{2}\times V_{k}+V_{k}\times V_{h}\right)\times (1/4{)}^{2}$$

where $E_{k}$ and $E_{h}$ are the mean of k and h with $V_{k}$ and $V_{h}$ being their respective variance. Next, the standard errors of the fin-body ratio were calculated and used to estimate CI widths at 0.95 significance level.

To estimate effect sizes at given percentage of change (??), an artificial dataset was generated by altering the coefficient of interest while maintaining other coefficient as well as y residuals at given x values. N data points were drawn with replacement from each dataset for calculation of kinematic parameters, which was repeated 200 times to generate distributions of parameters of interest. Effect sizes were determined using Cohen’s d:

$$ES=\frac{\left|\mu_{\mathrm{sim}}-\mu_{\mathrm{ori}}\right|}{\sigma }$$

where $\mu_{\mathrm{sim}}$ and $\mu_{\mathrm{ori}}$ are the mean of parameter values calculated from respective datasets and σ is the standard deviation of all 400 calculated parameters. The whole process was repeated for 20 times to estimate the mean effect size at given sample size (N) and percentage of change. To reduce program execution time, we used a fixed 40 ms before time of peak speed as the time of max angular velocity for fin-body ratio calculation. Other kinematic parameters were calculated as described above.

#### Statistical analysis of kinematic parameters among zebrafish strains

One-way ANOVA Tukey’s HSD test was used to compare parameters among zebrafish strains in Figure 7. All p values reported are adjusted p values.

## Supplementary Material

1

2

3

4

5

## Figures and Tables

**KEY RESOURCES TABLE:** 

REAGENT or RESOURCE	SOURCE	IDENTIFIER

Deposited data		

Behavior data	OSF	10.17605/OSF.IO/WJH25

Experimental models: Organisms/strains		

SAT zebrafish	Zebrafish International Resource Center	ZL1941
AB zebrafish	Zebrafish International Resource Center	ZL1
*Drosophila melanogaster* w^1118^	Bloomington Drosophila Stock Center	5905
*Caenorhabditis elegans*		N2

Software and algorithms		

Behavior software	OSF	10.17605/OSF.IO/WJH25
Analysis code	OSF	10.17605/OSF.IO/WJH25
Visual Studio Code	Microsoft	NA
Vision Develoopment License (Image Processing)	National Instuments	778044-35
Vision Acquisition License (Image Acquisition)	National Instuments	778413-35
LabVIEW Runtime Engine	National Instuments	LabVIEW Runtime

Other		

Computer RAM (64GB)	Amazon	B0884TNHNC
Computer case	Amazon	B08BF8YMXC
Computer Motherboard Mini-ITX, AM4 CPU slot, on-board NIC	Amazon	B089D34SZT
Computer SSD 1TB	Amazon	B08V83JZH4
Computer CPU with embedded GPU (AMD Ryzen 7)	Amazon	B091J3NYVF
Computer CPU fan Noctua	Amazon	B075SG1T3X
Computer power supply (450 W)	Amazon	B07JFLNG3B
Computer USB card	Amazon	B08B5BNZQ6
SM2 condenser holder	ThorLabs	SM2L05
Condenser and diffuser for IR	ThorLabs	ACL5040U-DG6-B
Tube to distance condenser from LED	ThorLabs	SM2L20
IR module holder	ThorLabs	SM2RC
IR LED SM2 to SM1 adapter	ThorLabs	SM1A2
Heatsink housing tube	ThorLabs	SM1M10
Heatsink to SM1 adapter	ThorLabs	SM1A6FW
Camera to SM1 adapter	ThorLabs	SM1A10
SM1 tube to imaging lens adapter	ThorLabs	SM1A9
IR filter	ThorLabs	FGL830
Camera module holder	ThorLabs	SM1RC
SM1 lens extension tube	ThorLabs	SM1-L03
Imaging chamber holder	ThorLabs	FP01
Post holders for chamber holder and IR module	ThorLabs	PH1
Posts for chamber holder and IR modulePosts for	ThorLabs	TR1
Post holder for camera module	ThorLabs	PH1.5
Post for camera module	ThorLabs	TR1.5
1/4-20" screws to attach post-holder to breadboard	ThorLabs	SH25S038
1/4-20" low-profile screws for enclosure	ThorLabs	SH25LP38
IR LED 12 V 2 A power supply	Amazon	B00Q2E5IXW
IR LED 940 nm 2.6 V Opulent LST1-01F09-IR04-00	Mouser	416-LST101F09IR0400
Ohmite heat sink	Mouser	SV-LED-325E
HexaTherm tape (attach LED to heatsink)	LEDSupply	A001
BuckBlock 1A	LEDSupply	0A009-D-V-1000
12V 1A power supply for daytime lights	Amazon	B00FEOB4EI
SMD5050 6500K white LED 12V light strip 60LED/meter (use 3 LEDs per apparatus)	Amazon	B075R4X1XL
Camera IMX174 USB 3 interface	Basler	acA1920-155um
Lens 50 mm VIS-NIR	Edmund Optics	67-717
Breadboard	Base Lab Tools	SABCUST
Rails for enclosure (see measurements)	Base Lab Tools	X2020-CUST
Hardboard for enclosure walls (see measurements)	Base Lab Tools	X2020-HB-CUST
Right angle joiner for LED strip	Base Lab Tools	X2020-AB1
Joiner cube for enclosure	Base Lab Tools	X2020-C3W
Spring-loaded t-nuts	Base Lab Tools	X2020-DTSB-M5-P10
M5-0.8 × 8mm Screws	Amazon	B07H18YDYB

## Highlights

- SAMPL is a scalable solution to measure posture and locomotion in small animals
- We use SAMPL to define balance and navigation behaviors in larval zebrafish
- SAMPL can resolve behavioral differences across genetic backgrounds
- SAMPL’s high resolution and throughput make it promising for behavioral screens
