# Supplementary material for: SAMPL is a high-throughput solution to study unconstrained vertical behavior in small animals
Source: Cell Rep. Author manuscript; Available in PMC 2023 Oct 23. (PMC10592459; doi:10.1016/j.celrep.2023.112573)
Supplement: 1 [file NIHMS1912773-supplement-1.pdf]

Cell Reports, Volume *42*

Supplemental information

SAMPL is a high-throughput solution to study unconstrained vertical behavior in small animals

Yunlu Zhu, Franziska Auer, Hannah Gelnaw, Samantha N. Davis, Kyla R. Hamling, Christina E. May, Hassan Ahamed, Niels Ringstad, Katherine I. Nagel, and David Schoppik

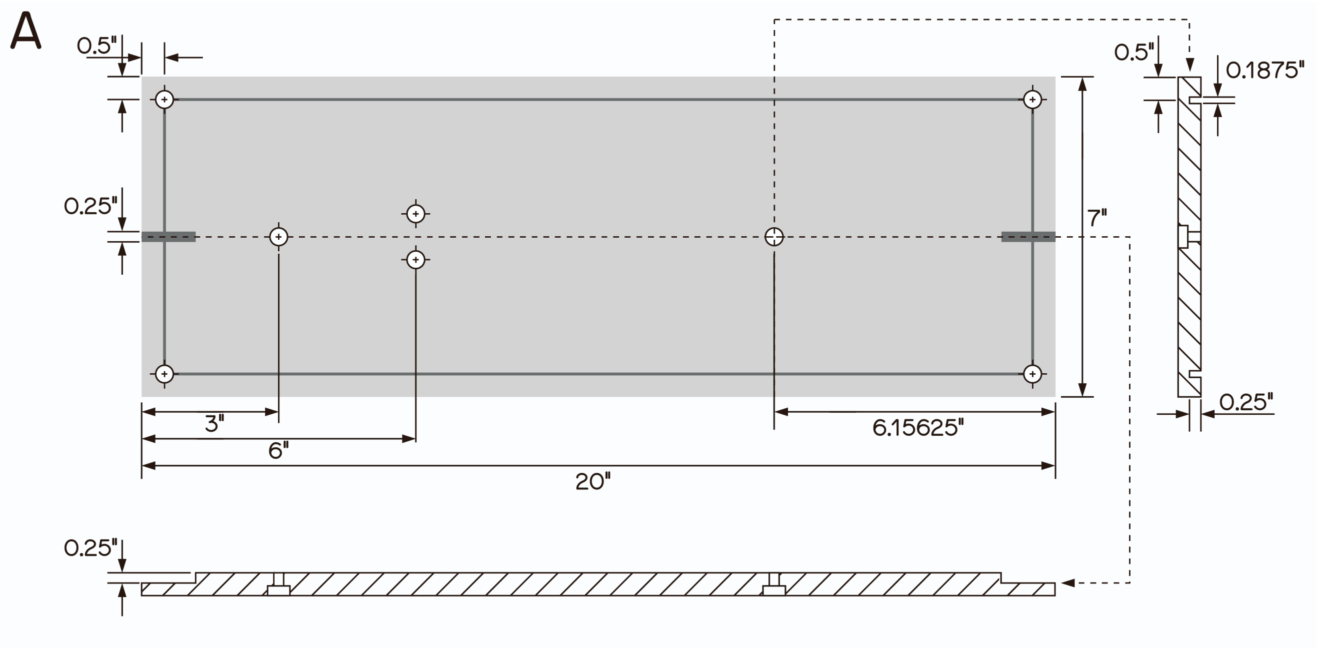

Figure S1: Custom breadboard for SAMPL base. Related to Figure 1.
(A) Custom aluminum breadboard, not anodized, 0.5" thick. All holes (8 total) counterbored for 1/4"-20 cap screw. Grooves to be cut on the side of the breadboard OPPOSITE to the counterbore.

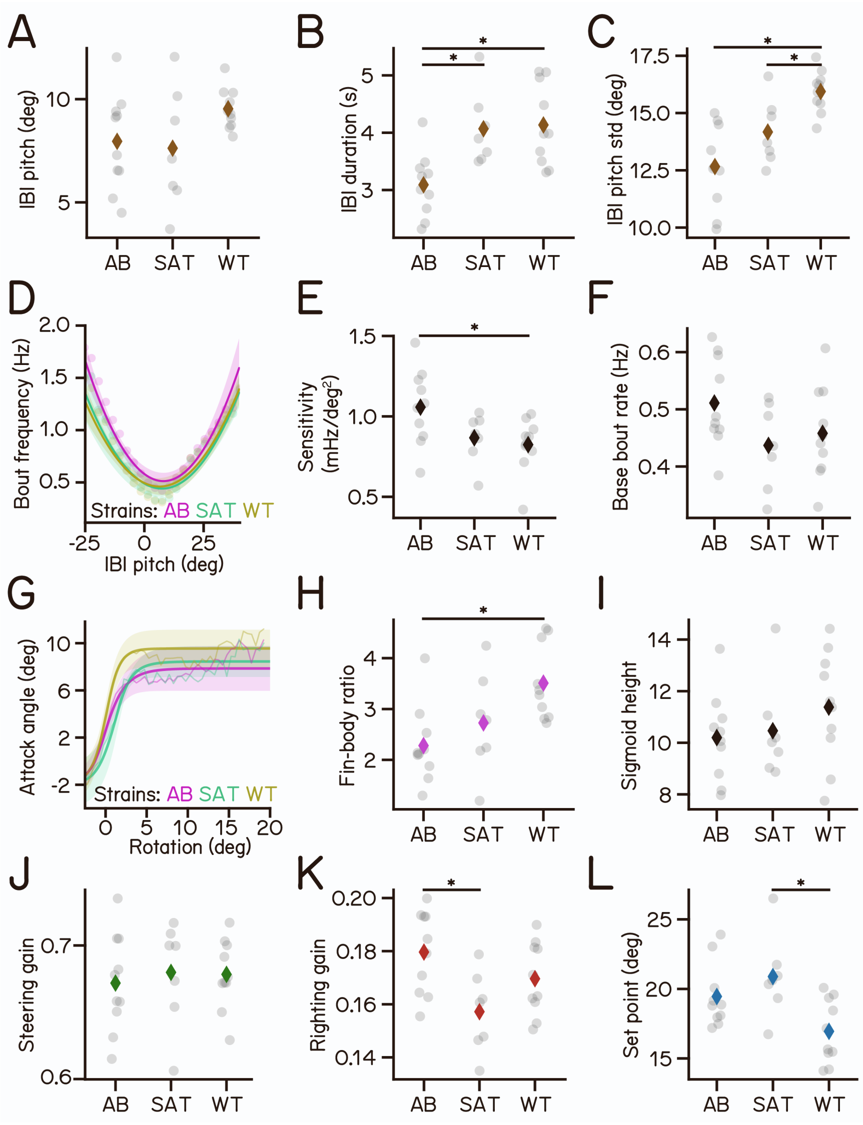

Figure S2: Variations of kinematic parameters among three different zebrafish strains. Related to "SAMPL can resolve slight variations in posture control strategies across genetic backgrounds"

(A) Average pitch angles during IBI.
(B) IBI duration (AB vs SAT p-adj = 0.0128; AB vs WT p-adj = 0.0034).
(C) Standard deviation of IBI pitch (AB vs WT p-adj = 0.0001; SAT vs WT p-adj = 0.0479).
(D) Bout frequency plotted as a function of IBI pitch modeled with parabolas.
(E) Sensitivity to pitch changes (AB vs WT p-adj = 0.0319).
(F) Baseline bout rate.
(G) Attack angles plotted as a function of body rotations modeled with sigmoids.
(H) Fin-body ratio (AB vs WT p-adj = 0.0066).
(I) Height of the sigmoid in **G**.
(J) Steering gain of different strains.
(K) Righting gain of different strains (AT vs SAT p-adj = 0.0133).
(L) Set point (SAT vs WT p-adj = 0.0094). For each strain of AB/SAT/WT, N = 10/7/10 repeats, n = 62457/27990/31532 bouts and 55683/25964/27946 IBIs from 225/117/195 fish. All reported p-values are adjusted p-values from one-way ANOVA with post-hoc Tukey HSD test.

Table S1: List of parts, prices per 12/2022. Related to Figure 1.

**Computer & software licenses ($2,300; one computer runs three apparatus)**	
RAM (64GB)	Amazon B0884TNHNC
Case (small form factor)	Amazon B08BF8YMXC
Motherboard (Mini-ITX, AM4 CPU slot, on-board NIC)	Amazon B089D34SZT
Solid state hard drive (1TB)	Amazon B08V83JZH4
CPU w/embedded GPU (AMD Ryzen 7)	Amazon B091J3NYVF
Quiet CPU fan (Noctua)	Amazon B075SG1T3X
Power supply (450W)	Amazon B07DTP6SLJ
USB card	Amazon B08B5BNZQ6
Operating system (Windows 10 Professional)	Amazon B00ZSHDJ4O
Vision Development License (Image Processing)	National Instuments 778044-35
Vision Acquisition License (Image Acquisition)	National Instruments 778413-35
Software Runtime Engine	NI LabView Runtime (free download)

**Shelving unit for 12 apparatus, ($2,100)**	
KVM switch to share keyboard, mouse and monitor w/cables	Amazon B001V9LQ52
Monitor 1920x1080	Amazon B07F8XZN69
Keyboard	Amazon B00CYX26BC
Mouse	Amazon B087Z733CM
Mobile wire shelving unit w/4 shelves 36"x81.5"x24"	McMaster Carr 2563T336
Extra shelf (handy to hold UPS and network gear up top)	McMaster Carr 5101T497
Uninterruptible power supply	Amazon B078D6KZ98
Spare battery for UPS (handy to have around)	Amazon B010XF8SCI
Timer (for light/dark)	Need 4, Amazon B005MMSTNG
Power strip (6', higher shelves, 2pk)	Amazon B082DVCCDR
Power strip (12', lower shelves, 2pk)	Amazon B08KZGT258
Wire ties (cable management)	Amazon B096ZHHRC3
Network cables CAT6a 10G 7ft (5pk & 10pk)	Amazon B01BGV2T5U
Network switch (Netgear GS110MX)	Amazon B076642YPN

**Networked data storage ($3,800)**	
500GB solid state drive for data server caching	Need 2 Amazon B07M7Q21N7
Data server Synology DS1621xs+	Amazon B08HYRYLPS
16TB Hard drives for data server. Order 7 (6+1spare)	Need 7 Amazon B07SPFPKF4
10GB NIC for data server	Amazon B07G9N9KJT

**Enclosure (BaseLabTools/Amazon/MetalsCut4U, $375 per apparatus)**	
Breadboard (see image w/measurements)	SABCUST
Rails for enclosure (see measurements)	X2020-CUST
Hardboard for enclosure walls (see measurements)	X2020-HB-CUST
Right angle joiner for LED strip	Need 2 X2020-AB1
Joiner cube for enclosure	X2020-C3W
Spring-loaded t-nuts (10pk)	X2020-DTSB-M5-P10
M5-0.8 x 8mm Screws	Amazon B07H18YDYB
Top: G90 galvanized steel (7.25in x 20.25in x 3in, 20 Ga.)	Tray, MetalsCut4U

**ThorLabs parts ($550 per apparatus)**	
Holds condenser	SM2L05
Condenser/diffuser for IR light	ACL5040U-DG6-B
Tube to distance condenser from LED	SM2L20
Adapts IR light holder to post	SM2RC
Adapts SM2 tube to SM1 tube	SM1A2
Tube to hold heatsink	SM1M10
Adapts heatsink / LED to SM1 tube	SM1A6FW
Adapts camera to SM1 tube	SM1A10
Adapts SM1 tube to imaging lens	SM1A9
Filter to pass only IR light	FGL830
Adapts camera/lens to post	SM1RC
Holds filter / allows camera/lens mounting	SM1L03

Holds imaging chamber	Need 2 FP01
Post-holder for chamber holder / IR assembly	Need 3 PH1
Posts for chamber holder / IR assembly	Need 3 TR1
Post-holder for camera/lens	PH1.5
Post for camera/lens	TR1.5
1/4-20" screws to attach post-holder to breadboard	SH25S038
1/4-20" low-profile screws for enclosure	SH25LP38

IR LED (assembly required, $100 per apparatus)

12V 2A power supply for IR	Amazon B00Q2E5IXW
XT60H connector for IR lights	Amazon B09ST768W2
940nm 2.6V IR LED Opulent LST1-01F09-IR04-00	Mouser 416-LST101F09IR0400
Thermal epoxy (attach heatsink to ThorLabs SM1A6FW	Amazon B08Z73HH23
Ohmite heat sink	Mouser SV-LED-325E
HexaTherm tape (attach LED to heatsink)	LEDSupply A001
BuckBlock 1A	LEDSupply 0A009-D-V-1000

Daylight LED, ($50 for three apparatus)

12V 1A power supply for daytime lights (5pk)	Amazon B00FEOB4EI
SMD5050 6500K white LED 12V light strip 60LED/meter	Amazon B075R4X1XL
DC power pigtail (to connect LED strip to power)	Amazon B0768V9V5Q
T tap connectors	Amazon B085XGYW1B

Imaging, ($1,200-$1,800 per apparatus)

Camera (IMX174 chip, USB 3 interface)	e.g. Basler acA1920-155um
Lens (50mm, VIS-NIR coating)	Edmund Optics 67-717
USB cable	e.g. Edmund Optics 86-770

Chambers, laser cut by Pololu ($200)

Chamber sides	12mm (10.2 - 12.75mm) #2025 black cast acrylic, opaque
Chamber faces	1.5mm (0.8 - 2.1mm) clear cast acrylic
Weld-On 4 acrylic cement & applicator	Amazon B00TCUJ7A8

Table S2: Recording parameters for different organisms. Related to Figure 2.

	Zebrafish $\leq$ 12 dpf	Zebrafish $>$ 12 dpf	*Drosophila*	*C. elegans*
Body low	14	14	100	20
Body high	255	255	255	255
Head low	45	45	30	21
Head high	255	255	255	255
Initial cut low	25	25	45	3
Initial cut high	120	120	145	30
Size low	180	250	80	30
Size high	260	450	180	80

Table S3: Measured parameters of posture and locomotion across development. Related to Figures 3-6.

Parameter	Unit	4 dpf	7 dpf	14 dpf	Format	Definition
Peak speed	mm/s	10.42 (3.85)	13.02 (4.99)	11.41 (4.20)	Mean of bouts (SD)	Peak speed of swim bouts
Initial pitch	deg	5.21 (31.49)	0.77 (21.81)	0.54 (18.64)	Median of bouts (IQR)	Pitch angle at 250 ms before the peak speed
Pitch at peak speed	deg	9.74 (29.16)	6.84 (20.35)	4.36 (19.73)	Median of bouts (IQR)	Pitch angle at time of the peak speed
Post-bout pitch	deg	10.57 (23.86)	10.21 (16.70)	6.88 (16.05)	Median of bouts (IQR)	Pitch angle at 100 ms after the peak speed
End pitch	deg	10.85 (23.59)	10.78 (16.79)	7.56 (15.55)	Median of bouts (IQR)	Pitch angle at 200 ms after the peak speed
Bout trajectory	deg	12.29 (27.52)	8.92 (20.19)	7.85 (22.89)	Mean of bouts (SD)	Peak trajectory, tangential angle of the trajectory at the time of the peak speed
Bout displacement	mm	1.12 (0.63)	1.36 (0.64)	1.35 (0.70)	Mean of bouts (SD)	Average displacement of fish during a bout when speed is greater than 5 mm/s
Inter-bout interval	s	1.78 (2.61)	1.89 (2.75)	2.13 (2.80)	Median of bouts (IQR)	IBI, duration between two adjacent swim bouts
Bout frequency	Hz	0.56 (0.70)	0.53 (0.69)	0.47 (0.59)	Median of bouts (IQR)	Frequency of swim bouts determined by the reciprocal of inter-bout interval
IBI pitch	deg	8.75 (17.73)	8.06 (13.07)	6.08 (11.24)	Mean of bouts (SD)	Mean pitch angle during inter-bout interval
IBI pitch standard deviation	deg	17.48 (1.60)	12.66 (1.80)	11.23 (1.28)	Mean of repeats (SD)	Standard deviation of IBI pitch, a measurement of stability
Sensitivity	mHz/deg^2	0.61 (0.18)	1.06 (0.23)	1.31 (0.34)	Mean of repeats (SD)	Sensitivity to pitch changes. Determined by the coefficient of the quadratic term of the parabola model for bout timing
Baseline bout rate	Hz	0.51 (0.06)	0.51 (0.08)	0.47 (0.11)	Mean of repeats (SD)	Y intersect of the parabola model for bout timing
Trajectory deviation	deg	5.46 (14.53)	4.13 (11.57)	4.35 (16.39)	Mean of bouts (SD)	Deviation of bout trajectory from initial pitch
Steering rotation	deg	2.30 (7.51)	3.00 (7.33)	1.94 (6.31)	Mean of bouts (SD)	Change of pitch angle from initial (250 ms before) to the time of the peak speed
Steering gain	-	0.64 (0.04)	0.67 (0.04)	0.51 (0.05)	Mean of repeats (SD)	Slope of best fitted line of posture vs trajectory at the time of the peak speed
Steering-related rotation	deg	1.72 (6.15)	1.74 (5.95)	0.99 (5.42)	Mean of bouts (SD)	Change of pitch angle from initial to the time of max angular velocity
Attack angle	deg	4.10 (16.16)	0.77 (9.68)	0.91 (5.25)	Median of bouts (IQR)	Deviation of bout trajectory from pitch at time of the peak speed
Peak angular velocity time	ms	50.60 (7.62)	39.16 (4.96)	50.00 (5.12)	Mean of repeats (SD)	Time of peak angular velocity in ms before time of the peak speed
Fin-body ratio	-	3.41 (0.86)	2.27 (0.76)	3.55 (1.98)	Mean of repeats (SD)	Maximal slope of best fitted sigmoid of attack angle vs early rotation
Sigmoid height	deg	16.47 (2.31)	10.28 (1.78)	25.15 (4.68)	Mean of repeats (SD)	Height of best fitted sigmoid of attack angle vs early rotation
Righting rotation	deg	0.92 (3.49)	2.64 (3.55)	1.90 (3.01)	Mean of bouts (SD)	Change of pitch angle from time of the peak speed to post bout (100 ms after peak speed)
Righting gain	-	0.15 (0.02)	0.18 (0.02)	0.18 (0.02)	Mean of repeats (SD)	Numeric inversion of the slope of best fitted line of righting rotation vs initial pitch
Set point	deg	13.00 (2.10)	19.47 (2.28)	13.60 (1.78)	Mean of repeats (SD)	X intersect of best fitted line of righting rotation vs initial pitch

SUPPLEMENTAL VIDEO

Movie S1

Movie S1. Stop motion instruction for box assembly. Related to Figure 1.

Movie S2

Movie S2. Example of recorded epochs of a fly, a shrimp, and a worm. Scale bar: 2 mm. Related to Figure 2.

Movie S3

Movie S3. Top: example of a recorded epoch of a freely-swimming zebrafish larva using the apparatus. Bottom: swim speed and pitch angles plotted as a function of time. Scale bar: 1 mm. Related to Figure 2.

Supplemental notes

Note S1. Hardware Design Principles

Camera

At the time of writing, the best price/performance ratio when using infrared light are the Sony Exmor line of complementary metal-oxide-semiconductor (CMOS) sensors. Sensors in the Exmor line are usually released as pairs, with a low-cost low-speed version of the same sensor available at the same time as a more expensive high-speed version. Our initial designed used the lower-cost IMX249 sensor; we have since switched to the faster IMX174 variant. These two sensors have a particularly large pixel size (5.86um), low noise (7e-), and a large well depth (32,513e-) allowing for exceptional dynamic range (73dB) and signal-to-noise ratio (45dB) at high-definition resolution (1936 × 1216 pixels). Quantum efficiency >900nm (i.e. the infrared range we will use) is 10%. Sony has released new sensors in the Exmor line regularly, but the trend has been to release sensors with increasingly small pixels. Thus for our purposes, the performance of the IMX174 remains unmatched.

Machine vision cameras are available with different interfaces used to stream data to a computer. The major difference between interfaces is the bandwidth available to each. The two most common interfaces for machine vision cameras at the time of writing are Gigabit Ethernet (125MB/sec) and USB3.0 (500MB/sec after overhead). Currently, there are commercially-available cameras with higher bandwidth interfaces utilize 10-tap CameraLink (850MB/sec), 10 Gigabit Ethernet (1250 MB/sec), 4xCoaXPress 2.0 (6,250MB/sec), and PCIe x8 (7,000MB/sec). Running our preferred IMX174 sensor at full resolution and speed for 8-bit images only requires 380MB/sec. Thus, USB3.0's low cost and relative ubiquity made it the most attractive option for our apparatus.

There are a number of manufacturers that make cameras built around the IMX174 with a USB3.0 interface. Cameras from major manufacturers all conform to the GenICam standard making them largely interchangeable, particularly when using the Vision Acquisition software from National Instruments. We have successfully used cameras from Ximea (MC023MG-SY), Basler (acA1920-155um), and FLIR (GS3-U3-23S6M-C), others include SYS-Vistek (exo174CU3) and Daheng Imaging (MER2-230-168U3M). We have also used cameras ordered directly from different manufacturers – at a substantial discount – available via alibaba.com: Hangzhou Huicui Intelligent Technology Co. Ltd. (A7200MU130), Hangzhou Contrastech Co. Ltd. (Mars2300S-160um), Shenzhen Hifly Technology Co. Ltd. (MV-AU231GM). When ordering directly from manufacturers we specify Delivery At Place (DAP) shipping. The primary differences that we've encountered are whether a particular model implements binning or other on-camera computations, heat management, and different manufacturer-provided APIs. When we use multiple cameras from the same manufacturer on the same computer, we have also noticed that certain cameras will throw timeout errors on some USB ports but not others; shuffling cameras and ports has worked to solve this problem. At the time of writing, supply chain issues mean that most major camera companies quote long lead times, but cameras ordered directly through alibaba.com all shipped within two weeks.

Illumination

Image quality is proportional to available light. Further, the size of the illuminated area defines the size of the field that can be imaged. Finally it is imperative for our experiments that from the fish's perspective that the "dark" period is completely dark. We therefore chose 940nm LEDs as our source of infrared illumination. This left us with three options to build our illumination source: LEDs mounted on adhesive strips, "star" style LEDs with 1-4 dies on a single PCB, and a high-power LED array. The LED strips had too little illuminance for our purposes due primarily to the spacing of the LEDs. The high-power LED array had ample illuminance but generated so much heat that it required active cooling.

We developed a simple illumination module to provide diffuse IR light across a 50mm circle An LED mounted on a "star" PCB (Opulent LST-01F09-IR04-00, Mouser) provided ample light. We mount each "star" LED with thermal adhesive to a small heat sink (Ohmite SV-LED-325E) which in turn is glued to a Thorlabs adapter (SM1A6FW) to allows the wires to exit and the LED/heatsink to connect to collimation and diffusion optics. The heat sink is machined (either with a Dremel hand-held tool or a mill) on one side to allow the wires that power the LED to lie flat against the heatsink. We power multiple illumination modules in series using a constant current LED driver (LuxDrive BuckBlock 1000mA). Our illumination setup generates negligible heat and our modules run continuously for years.

Our imaging parameters are fixed across experiments and optimized to give the highest quality data we can achieve with our hardware. The gain of the camera is set either to its lowest value or just above to minimize noise. Our exposure time is either 750 µsec or 1msec, allowing for a

crisp image in the face of the fastest movements that fish can make. The illuminated area is circular, but the image sensor size is rectangular. We therefore crop the sides of the image to produce a square that fits within the illuminated area.

Lens

Our choice of lens was guided by the need to balance different demands:

1. The longer the working distance, the greater the space needed between the sample and the lens. We wanted our apparatus to fit length-wise on a 24 inch shelf, and so we needed to minimize the working distance.
2. The entire depth of the tank needs to be in focus, but not beyond that because we'd like to blur our LED.
3. The lens should be coated to pass IR light
4. The lens should be easy to mount to the base of the apparatus; mounting the lens instead of the camera allows drop-in replacement of cameras from different manufacturers, which have different positions of the tripod mount relative to the sensor.
5. The lens should have a simple way to mount an IR-pass filter (e.g. common thread).

Unfortunately, we were not able to find a single lens that met all of these criteria. Instead, we adapted a 50mm (Edmund Optics 67717) lens by placing a small Thorlabs tube (ThorLabs SM1-L03) between the lens and the camera. We mounted a 25mm IR pass filter (ThorLabs FGL830) inside the Thorlabs tube. By moving the lens farther from the sensor we decreased the minimum working distance sufficiently. Finally, the Thorlabs tube allows us to mount the lens to the breadboard directly.

Behavioral arena

To maximize the amount of time the fish swam in a plane orthogonal to the camera, we used rectangular chambers. Initially we chose glass colorimeter cuvettes (Starna Cells Inc, Atascadero CA): they are made of an inert material (glass) and come in a variety of sizes. Due to supply chain issues, we switched to custom-fabricated chambers, plans attached. We now assemble these from laser-cut acrylic, cementing a front and back side to a u-shaped piece that forms the other sides. These chambers are considerably cheaper and less prone to breakage than glass and can be rapidly modified to allow for different experiments.

Enclosure

We designed a custom aluminum base with tapped holes for post-holders for the IR LED, chamber holder, and camera/lens/filter holder. We used custom-cut extruded aluminum rails to frame the sides and top. The sides are made of black foam-core sized to fit in grooves in the breadboard and rails. The top rails have a cross piece that holds the LED strip used to provide circadian lighting. All parts are fabricated to order by Base Lab Tools Inc (Stroudsburg PA). The top is a steel tray fabricated to order by MetalsCut4U (Avon Lake OH). Our current enclosure took roughly three months to prototype before settling on the final design.

Shelving and fleet organization

We have organized our fleet of apparatus to sit on mobile wire shelving. Currently, we use 36 × 24" × 81.5″ adjustable wire shelving units (McMaster Carr, Robbinsville NJ). We prefer to have the shelving on casters as it makes accessing the back of the units considerably easier. Shelving is organized such that one computer and three apparatus sit on a single shelf. Enclosures on a given shelf are color-coded (blue, gold, and red) so that each apparatus can be uniquely identified by a color/shelf/module combination; this also facilitates wire labeling. Each shelf has its own power strip that controls the computer, the IR lights, and the white LEDs; all strips plug into a single uninterruptible power supply (APC SmartUPS 1000C).

Our aim in specifying module size was to ensure that multiple investigators could set up experiments simultaneously, and to minimize the cost One unit has four shelves so that a single "module" consists of four computers and twelve apparatus. Each module has a dedicated monitor/keyboard/mouse on an adjacent desk, shared by the four computers using a KVM switch (IOGEAR GCS1794). A module has its own dedicated unmanaged Ethernet switch (NETGEAR GS110MX) that allows Gigabit speed communication between computers and 10 Gigabit speed between modules.

Computer hardware

Computer hardware was chosen to ensure adequate performance while minimizing cost, noise, and size. We found that building our own computers was the only path forward in the face of supply chain issues and strict optimization criteria. We opted to build around what was, at the time of writing, the previous generation of AMD microprocessors (Ryzen 7 5700G) cooled by a Noctua NH-L9a-AM4 fan (to minimize acoustic noise). We chose a Mini-ITX form factor motherboard that allowed us to use a small case (Cooler Master NR200). Other parts (64GB RAM, SSD, power supply) were chosen based on availability; a full parts list is attached (**Table S1**). We recommend using https://www.pcpartpicker.com to minimize cost and ensure compatibility of different components. All computers run Windows 10 Professional (Microsoft, Redmond WA).

Note S2: Acquisition Software Design Principles

What we don't measure

To extract the maximum amount of useful information about posture and locomotion with the minimum amount of overhead we had to be selective about what we measure. Our imaging field is located in the center of the arena; fish that swim at the bottom, top, or sides of the tank where there is a boundary are excluded from tracking. While multiple fish swim in the same arena, we do not take data when more than one fish is in the imaging field to sidestep the need to track fish identity. Our arena is sized to allow fish to swim freely but its shape (a rectangular solid) encourages fish to swim in line with the imaging plane; we exclude frames where fish turn away from the field of view (i.e. are swimming toward/away from the camera). Finally, capturing the full range of rapid propulsive undulations of the fish tail requires a frame rate of 500Hz-1kHz.[73,130] As changes to posture and locomotion are much slower, we opted to record at 160Hz. Together, these choices allowed us to optimize our algorithms to achieve the speed necessary to process video in real-time.

Algorithms to measure posture and position

Our apparatus extracts the position and pitch orientation of zebrafish in real-time over days using a simple set of common machine vision processing steps:

1. Measure the absolute difference between the current frame and the background (fish-free) image.
2. Threshold the difference image such that all small differences are set to zero.
3. Dilate the image three times in succession to remove any larger clumps that are still smaller than a fish.
4. Extract and quantify all particles in the image.

Real-time video processing allows efficient data extraction during video acquisition. Our design of the architecture is further discussed in the section: Optimizations for speed.

Below we detail a number of additional processing and optimization steps to ensure that we maximize useful data.

Measuring the pitch of the fish

To extract the pitch (the angle of the fish with respect to the horizon), we perform the following steps to ensure that the sign and magnitude of the angle is correctly assigned:

1. Fit the particle with an ellipse and extract the angle of the long axis with respect to the horizon.
2. Threshold the original difference image again to identify the pixels that correspond to the head of the fish.
3. Using the head and body (X,Y) coordinates determine whether the fish was facing to the left or right.
4. Assign the correct angle and sign such that nose-up posture is always positive and nose-down is always negative.

These steps ensure that the data saved follows a simple and intuitive convention for posture.

Optimizations for speed

To optimize our code for speed, we use a set of thresholds to rapidly evaluate and reject frames

1. Before any processing, we sum the pixel values in the frame. If it is too low (no fish in frame) or too high (more than one fish in the frame) we reject the frame.
2. After the particles are identified we reject the frame if a particle is touching the edge (fish partially out of frame), if there is more than one particle (multiple fish) or if the length of the particle is too short (fish bending in/out of the field of view). We define an epoch as a set of continuous frames that pass all our exclusion criteria (i.e. that contain a single fish in frame). Epoch duration is tracked and, when too short, can be rejected.

In addition to optimizing the algorithm, we adopted a producer-consumer architecture to decouple video acquisition from video processing and saving data. Our software runs two routines: the "producer," which acquires frames from the camera and places them in a queue in memory, and the "consumer" that extracts each frame from the queue and processes it in turn. Our program monitors the size of the consumer buffer and, if it has less that 10% free, pauses the producer routine for 15 s to allow the buffer to clear. In this configuration, the performance ceiling shifts from CPU speed (i.e. how quickly can a frame be processed) to the amount of RAM available (i.e. how many frames can be queued). At the time of writing this, doubling the amount of RAM is considerably less expensive than doubling CPU performance. The choice of architecture thus brings down the cost of the computer.

Saving raw video

While the bulk of our experiments rely on real-time processing of video it is often useful to save the actual data. Further, we wanted to be able to set user-defined criteria to determine in real-time which videos were worth saving. Leveraging the producer-consumer architecture, our software contains a routine that independently buffers the frames being analyzed and, if, the video to be saved meets user-defined criteria, will pass the frames to an independent program to write them to disk. For example, we can ensure that the video to be saved is of a certain length. Similarly, we can filter the video images[131] to determine if the target is in crisp focus (useful for larger arenas, or higher magnification) and only save high quality videos. By separating video writing from acquisition and processing, comparatively slow operations such as video compression and/or saving video to a network-accessible shared drive do not compromise performance.

Apparatus control software

Our algorithm relies on common and mature image processing routines and could be instantiated in any modern programming language. Since we had run this algorithm for the better part of a decade we were confident that it was sufficiently stable to compile into a distributed executable, which would greatly simplify deployment to a fleet of apparatus. Our original implementation was written in LabVIEW (National Instruments, Austin TX) which was stable and accommodated all the lab's hardware changes for the past decade. We therefore opted to update the LabVIEW code, which we distribute both as source and executable versions. Running the executable requires each computer to have the LabVIEW Runtime Engine (free download) installed, as well as a license for NI Vision Acquisition software (NI 778413-35) and the Vision Development Module Run-Time engine (NI 778044-35).

User interface

We designed the interface to enable easy initialization of experiments, rapid graphical and quantitative visualization of video processing and performance, and to minimize error. Launching the executable starts the program, which allows the user to fill out various text, numeric, and drop-down fields that describe the experiment. The user then monitors the video feed until no fish are in frame and then selects that image as the background. We have found that this initial bit of monitoring both compensates for slight day-to-day differences in arena placement. More importantly, it forces the user to monitor the live feed at the beginning of each experiment, a useful bit of mindfulness that minimizes lost data. Once running, the user can: monitor the output of each step in the processing algorithm graphically, monitor the number of times the consumer buffer has overflowed (usually zero), update the text fields, and stop the program. Hardware parameters are stored in a text file that can be easily edited directly. Experiment parameters are similarly saved to text files and can be reloaded to save time.

We have implemented a number of user interface items to minimize confusion in the face of a fleet of instruments. First, we have color-coded versions of the executable (blue, gold, and red) where the background clearly differentiates the version. Each version has its own configuration file that, during setup, is coded to a particular apparatus. Thus the user is always aware of which apparatus they are interfacing with based on color cues. Next, we added a “debug” button to the front panel that allows for direct monitoring and editing of all program variables. In “debug” mode the user has the option to save raw video.

Note S3: Hardware assembly guide

In this Appendix, we walk through box assembly and recording settings. Refer to **Note S5** for executing experiments and SAMPL data analysis.

Hardware assembly

Our design of hardware allows connecting up to three SAMPL boxes to one computer while using one set of power supplies (for IR LED and daylight LED). See **Video S1** for video instruction on box assembly.

1. Camera module
    a. Attach 1 × 1.5 inch post (TR1.5) to the camera module holder (SM1RC). Screw in tightly.
    b. Assemble lens and camber. Sequentially connect parts below: camera lens, SM1A10 adapter, IR filter, SM1-L03 extension tube, assembled camera module holder, SM1A9 adapter, and the camera.
2. IR illumination module
    a. Attach wired IR LED to the heatsink and SM1A6FW adaptor (see below for instruction).
    b. Carefully mount the condenser into SM2L05 tube.
    c. Assemble the IR module by sequentially connecting parts below: IR kit, SM1M10 tube, SM1A2 adapter, SM2L20 tube, and the mounted condenser.
    d. Tightly attach TR1 post to SM2RC holder.
3. Chamber holders
    a. Take off rubber covers on the tip of the screws on the chamber holders (FP01).
    b. Mount holders onto TR1 posts using 8–32 screws.
    c. Assemble the IR module by sequentially connecting parts below: IR kit, SM1M10 tube, SM1A2 adapter, SM2L20 tube, and the mounted condenser.
    d. Tightly attach TR1 post to SM2RC holder.
4. Put together the box
    a. Mount one of the short rails between two long rails using right angle brackets and T-nuts.
    b. Mount the other two short rails onto the long rails using slotted cubes and low-profile cap screws (SH25LP38).
    c. Adjust the position of the middle rail so that is approximately 13 cm away from one end of the frame.
    d. Attach post holders to the base plate using cap screws (SH25S038). Note that the one for the camera module is the longer post holder (PH1.5).
    e. Mount 4 medium rails onto the base plate using standard cap screws.
    f. Insert all the modules onto the base plate. Connect USB cable to the camera.

g. Make a notch in the middle of the shorter side of a small panel and insert it between the rails on the side of the camera module.

h. Insert 2 large side panels.

i. Attach daylight LED to the top frame (see below for instruction).

j. Pass IR and daylight LED wires through the front notch of the baseplate.

k. Insert the front panel.

l. Attach the top frame.

IR light wiring

Solder 2x 9″ wires onto the IR LED “star.” Attach IR LEDs to heatsinks using HexaTherm tape. Note that in order to pass the wires through the heatsinks and the SM1A6FW adapter on the opposite end, the ears of the Ohmite heatsink need to be trimmed down a little. When done, attach the heatsink to the adapter using thermal epoxy. To simplify light wiring, we use one 1000 mA BuckBlock to dirve 3 IR lights in series for 3 boxes on the same level of the shelf. To do this, one needs 2 × 7" wires to connect adjacent IR cables and 1 × 22" wire connecting the further IR to the BuckBlock. Use another 8″ wire to connect the closest IR to the BuckBlock. We recommend using XT60H connectos to link these wires to the IR light cables and connect wires to the BuckBlock for the ease of troubleshooting and replacement. Finally, connect the BuckBlock to 12 V 2 A power supply through pigtail adaptors.

Daylight wiring

Each box uses a strip of 6 daylight LEDs. Our choice of daylight LED comes with double sided tape already attached to the back side of the LED which is used to install LED strips to the top frame. To wire daylight LED strips, solder 2 × 20" wires to the LED strip. Heat shrink sleeves can be used here to strengthen connections. Twist the wires at the end close to the LED. This helps with cable management in the box. Bend the cables 90° in the XY plane (perpendicular to the illumination direction) so that the wires won’t get into the field of view.

To simplify light wiring, we use one 12V 1A power supply to drive 3 LED strips in parallel. To do this, cut 1 × 27" wire for connecting the positive end of the DC plug to the LED strip. Prepare 3 wires for the negative end of the strips each measured 10", 18", and 27". Insert one end of all three wires for the negative end into the negative terminal of a pigtail connector, connect the other ends to the LED cables. For positive end, we recommend using T tap connectors (B085XGYW1B) which allows easy disconnection.

PC setup

Assemble computer parts. Make sure 1 PCI-e USB card is installed into each PC. Connect power cable and Ethernet cable. If desired, connect 3 cameras to three different USB BUS on the PC: specifically, one to a PCI-e USB card, one to a USB 3.0/3.1 port on the motherboard in the back of the PC, and one to a USB 3.0 port on the front panel. If desired, connect to the KVM switch.

Turn on the PC, setup Windows. If necessary, change settings below to achieve peak performance: select AMD High Performance in Power Settings; set Sleep time to Never; set hard disk sleep time to 0 in Advanced Power Settings.

Install behavior programs

We provide three executable programs (Blue, Gold, Red) that can run simultaneously on the same PC. Refer to the **key resources table** for access to the programs. To install executables, download `*.exe` files and corresponding configuration files (`* Configuration.ini`). Create a folder under `C:/`and move configuration files to `C:/Data/`. Install required NI software and activate: LabVIEW Runtime, Vision Acquisition, and Vision Runtime. Restart computer.

Open NI Max, rename cameras to `camBlue`, `camGold`, and `camRed`. Set camera settings.

- Field of view - X: left = 360; resolution = 1216
- Field of view - Y: top = 0; resolution = 1200

- Under Acquisition attributes - Receive time stamp mode = System time
- Under Camera attributes - Analog control - Gain = 1; Black level = 1 (if applicable)
- Under Acquisition Control - Exposure time = 1000; Trigger activation = Rising edge; Frame Rate = Freerun (for 166 Hz with our cameras of choice, or set to desired frame rate)

Open configuration files and set box number to desired values. We use box number as a unique identifier for different behavior boxes. Check camera name to make sure it's the same as the corresponding camera names in NI Max.

Open behavior programs, now that you should see images showing up on the preview windows.

Camera calibration

Once the apparatus has been assembled and software has been installed, align the field of view (FOV) to the center of the IR light circle. Raise or lower the post holding the camera module to center the FOV in Y and roll the module to level it.

Next, calibrate the scale of the FOV to 60 pixels/mm. To do this, secure a micrometer in a chamber and place it into the box. Snap a picture of it using NI Max, then measure the scale using the image of the micrometer. If necessary, loosen the SM2RC adapter and move the camera and lens forward or backward to achieve the correct scale.

Illumination adjustments should be completed with the behavioral arena in place. To calibrate exposure, first ensure the correct IR light is in use and set the aperture ring between f/16. In NI Max, the peak of the image histogram peak should be around 128 (the middle of the 8-bit range). If necessary, exposure can be reduced by lowering exposure time or increased by opening up aperture to f/11.

Network setup

We use a Synology data server as a repository to store behavior data. Hard drives are setup as RAID 10. Each SAMPL rack has its own ethernet switch, which can be connected to other switches as necessary.

Note S4: Data Analysis Software

In this appendix, we discuss algorithms for the data analysis and plotting software. We assume that the user is working with data from larval zebrafish here. If not, the specific parameters identified here are unlikely to translate as other organisms move differently but can nonetheless be used as a starting point. Refer to Note S5 for instruction for use. Refer to the **key resources table** for access to the code.

Read DLM files

Each SAMPL session (from `Start experiment` to `Stop`) generates one tab-delimited (i.e. `dlm`) file. Each time point appears as a row of tab-separated values in the `.dlm` file. Columns, from left to right, are time stamp, fish number in the field of view (FOV), pitch angle (0–90°), x coordinate for body, y coordinate for body, x coordinate for fish head, y coordinate for fish head, raw fish angle (0–180°), epoch number, and estimated fish length.

Each `.dlm`'s data is loaded as a Pandas DataFrame for further analysis (see `src/SAMPL_analysis/preprocessing/read_dlm.py` for details). Each raw DataFrame contains multiple epochs. An epoch is defined as duration when a fish is detected in the FOV. See Note S1 for details on the algorithm for animal detection.

Extract epochs

We calculated swim attributes, such as angular velocity, swim speed, instantaneous displacement, etc., from recorded pitch angles and fish body coordinates. To extract quality epochs from the recorded data, epochs are analyzed and passed through several quality control filters:

1. each epoch is truncated by 50 ms at both the start and the end to eliminate frames when fish is entering/exiting the FOV;
2. epochs with duration shorter than 2.5 s are excluded (for 1 & 2, see function `raw_filter()`);

3. epochs with frame drop greater than 3 frames are excluded;
4. epochs with direction of fish translocation opposite to where the head points toward are dropped (for 3 & 4, see function `dur_y_x_filter()`);
5. epochs with aberrant displacement jumps are excluded;
6. epochs with improbably large angular velocity greater than 250°/s or angular acceleration larger than 32000°/s^2 are excluded (for 5 & 6, see function `displ_dist_vel_filter()`).

All the processes above can be found in the script `src/SAMPL_analysis/preprocessing/analyze_dlm_v4.py`.

Get bout and inter-bout data

Epochs that pass the quality control are used to extract swim bouts using function `grab_fish_angle()` under `src/SAMPL_analysis/bout_ analysis/grab_fish_angle_v4.py`.

We use a swim speed threshold of 5 mm/s to determine swim windows. Adjacent swim windows with intervals smaller than 100 ms are combined. Next, we find the time of the peak speed for each swim window and extract frames in a range of 500 ms before to 300 ms after that. Inter-bout intervals (IBI) are determined as time between adjacent swim bouts with a 100 ms buffer window deducted from both the beginning and the end and IBI data is extracted accordingly. Baseline is considered the time during which larvae swim slower than 2 mm/s and baseline parameters are extracted accordingly.

Note that an epoch can only contain a single detected fish. The number of swim bouts extracted from an epoch various extensively depending on the quality of the epoch (and behavior of fish). Having too many fish in the chamber may lead to low yields of aligned bouts despite having a large number of epochs. For details of fish detection, refer to **Note S1**.

Export analyzed results

Numerous attributes are saved as DataFrames under `keys` in HDF5 format files using our analysis pipeline. Once the analysis is complete, three output data files are generated: `all_data.h5`, `bout_data.h5`, and `IEI_data.h5`.

The `all_data.h5` file contains epoch-based data including raw data from DLM files, epoch attributes, baseline angular velocity, etc. The `bout_data.h5` file includes bout attributes and aligned bout data such as pitch angles and speed. The `IEI_data.h5` file contains all inter-event interval (IEI) data, or IBI. Refer to `docs/` for a complete list of saved attributes and their description. In addition, a metadata table including recording frame rate, number of aligned bouts, and other information is generated and saved to the same directory.

All results are saved as “long format” DataFrames with each row representing a time point or a bout/IEI, depending on the type of the result (one value per time point vs. per bout/IBI). Values of multiple aligned bouts are stored in successive rows.

All functions above can be called with script `src/SAMPL_analysis/SAMPL_analysis.py`. Refer to **Note S5** for running instructions. For a record of analyzed files, frame rate, number of aligned bouts, etc., refer to the log file generated under `src/`.

Load analyzed data and calculate parameters

We include several plot functions under `src/SAMPL_visualization/` that calculate and plot all the parameters we report in the main text. These functions require an input of a root directory containing analyzed data. For recommended behavior data structure, see **Note S5**.

Once data is found, plot functions get frame rate from metadata files and calculate the index of time of peak speed which is used to calculate the number of aligned frames and initialize other constants. Note that plot functions only read one frame rate for all the data to be plotted. Therefore, make sure all experiments are done at the same frame rate. To combine results from different frame rates for plotting, extract parameters of interest separately for experiments with different frame rates and concatenate the results afterward. We only plot zeitgeber day data in this version of the code. Users may modify the `day_night_split()` function to extract zeitgeber night results if intended.

To load analyzed swim bouts and IBI, we loop through all subfolders under the root directory and read DataFrames from HDF5 files, extract and calculate desired parameters and concatenate results. Each plot function extracts parameters in different ways.

For time series values to be plotted as a function of time, data is loaded from the `all_data.h5` file. The key `prop_bout_aligned` contains propulsive bouts that have been aligned and `grabbed_all` includes all epochs that contain swim bouts. See `plot_timeseries.py` for examples.

Bout parameters, such as speed, displacement, pitch angles and attack angles, are also extracted from `prop_bout_aligned` key containing aligned swim bouts. We use a dedicated function for calculating these swim parameters: `extract_bout_features_v4()`. These parameters can be further used to get steering and righting gains. See `get_kinetics()` for more. Note that some parameters are determined by specific time points (such as initial pitch, post-bout pitch, etc.). To determine frames that are the closest to these time points, we use half round up for rounding.

IBI data is loaded from the `IEI_data.h5` file under key `prop_bout_IEI2`. For bout timing estimation, we calculate bout frequencies as reciprocals of bout intervals (IBIs). See `plot_bout_timing.py` and `plot_IBIposture.py` for examples.

To calculate fin-body coordination, users need to determine how the `rotation` is calculated. One way is to use `rotation to time of peak angular velocity` which requires estimation of time of peak angular velocity. To do this, we first calculate angular velocity using smoothed pitch angles and adjust the signs so that values are positive before time of the peak speed. Median of angular velocity time series from the same experimental repeat (see **Note S5** for data organization) is used to find time of peak angular velocity. Lastly, we average results across experimental repeats to determine the peak angular velocity time. However, this calculation requires a large amount of bout data. Alternatively, one may use a fixed value for time of peak angular velocity. Generally, we found −50 ms (50 ms before time of peak speed) to be a good value to use. Once the time of peak angular velocity is determined, rotation is calculated by pitch change from 250 ms before peak speed to time of peak angular velocity. Some scripts have the option to sample data from each experimental repeats. See **Note S5** for instruction.

Visualize results

We use the Seaborn package for data visualization.[132] Each plotting script generates a folder under `figures/` and saves figures as PDFs. Below is a list of available plotting functions and their descriptions. For more details, refer to the `README` document.

1. `plot_timeseries.py`

plots basic parameters as a function of time. Modify `all_features` to select parameters to plot. This script contains two functions: `plot_aligned()`, `plot_raw()`. Change variable `all_features` to select parameters to plot.

2. `plot_parameters.py`

plots swim parameter distribution and 2D distribution of parameters for kinetics calculation. This script contains function: `plot_parameters()`.

3. `plot_IBIposture.py`

plots Inter Bout Interval (IBI; aka inter-event interval, IEI) posture distribution and standard deviation. This script contains function: `plot_IBIposture()`. This script looks for `prop_Bout_IEI2` in the `prop_bout_IEI_pitch` data which includes mean of body angles during IBI. When input root directory contains multiple experimental repeats, the scripts allows sampling of IBIs from each repeat by specifying argument `sample_bout`.

4. `plot_IBIposture.py`

plots Inter Bout Interval (IBI; aka inter-event interval, IEI) posture distribution and standard deviation. This script contains function: `plot_IBIposture()`. This script looks for `prop_Bout_IEI2` in the `prop_bout_IEI_pitch` data which includes mean of body angles during IBI. When input root directory contains multiple experimental repeats, the scripts allows sampling of bouts from each repeat by specifying argument `sample_bout`.

5. `plot_bout_timing.py`

Plots bout frequency as a function of IBI pitch and fitted coefficients of function. This script contains function: `plot_bout_frequency()`. When input root directory contains multiple experimental repeats, the scripts allows sampling of bouts from each repeat by specifying argument `sample_bout`.

6. `plot_kinematics.py`

Plots righting gain, set point and steering gain. This script contains function: `plot_kinetics()`. When input root directory contains multiple experimental repeats, the scripts allows sampling of bouts from each repeat by specifying argument `sample_bout`.

7. `plot_fin_body_coordination.py`

Plots attack angle as a function of rotation and calculates fin-body ratio. Rotation is calculated by pitch change from −250 ms to −40 ms. This script contains function: `plot_fin_body_coordination()`. For reliable sigmoid regression, 6000+ bouts is recommended. When input root directory contains multiple experimental repeats, the scripts allows sampling of bouts from each repeat by specifying argument `sample_bout`.

8. `plot_fin_body_coordination_byAngvelMax.py`

Plots attack angle as a function of rotation and calculates fin-body ratio. Rotation is calculated by pitch change from −250 ms to time of max angular velocity. For reliable sigmoid regression, 6000+ bouts is recommended. When input root directory contains multiple experimental repeats, the scripts allows sampling of bouts from each repeat by specifying argument `sample_bout`.

Note S5: Standard operating procedure for running experiments and analyzing data with SAMPL

In this appendix, we provide a step-by-step instruction for running experiments and analyzing SAMPL data. Refer to the **key resources table** for access to SAMPL analysis and visualization scripts.

Experimental design

SAMPL experiments usually involve comparing behaviors of two or more groups of fish with different mutations, transgenic backgrounds, or manipulation. We suggest first deciding *a priori* on the total number of bouts required to resolve differences ?? with the desired power. Typically, one SAMPL experimental repeat containing two 24-h sessions using 3 boxes with 5–7 larvae per box yields 3000–6000 bouts, which is usually sufficient for parameter calculation Figure ?? However, multiple factors can affect data size per repeat, such as: manipulations (mutation/drug treatment), the throughput of manipulation, the availability of apparatus, and the number of larvae with desired background per clutch. We therefore suggest running a pilot experiment first to determine the number of bouts that can be expected per box. Once done, we suggest defining an "experiment" with respect to the desired number of bouts, which will specify the number of boxes and larvae per box required. Outlier boxes with too few or too many bouts (e.g. more/less than 2SD) can then be excluded from further analysis according to pre-determined criteria. Finally, we recommend running the full "experiment" multiple times to ensure that the findings are reproducible, and to report the variance across estimated parameters. Certain circumstances may be ill-suited to this approach: for example, if particular genotypes of larvae are especially rare, such as in the case of doubly biallelic mutants, or genotypes that simply swim drastically less. In such cases one can combine swim bouts across experimental repeats, and report the estimated error in pararmeter estimates using statistical resampling techniques such as the jackknife.

Running an experiment

One typical SAMPL experimental repeat contains two 24-h sessions. We suggest running zebrafish larvae at one of 3 time points: 4–6 dpf, 7–9 dpf, or 14–16 dpf. Larvae should be given 30 min of access to food before being placed into chambers. We suggest putting 5–8 larvae into one standard chamber and 1–3 larvae in one narrow chamber to maximize data yield. Behavior recording requires having a single fish in the FOV at a time. Appearance of additional larvae will disrupt fish detection. We suggest transferring 25–30/10-15 mL E3 medium into each standard/narrow

chamber to account for evaporation and maximize likelihood of fish swimming in the FOV. Throughput of the apparatus can be found in Figure 2 (standard chamber based on 5–8 larvae; narrow chamber based on 2–3 larvae).

With SAMPL, one computer can control up to three behavioral apparatus, or "boxes." Once the fish chamber is put into the box and secured, open the program (Blue, Gold, Red) corresponding to the box to run on the computer controlling the boxes. Enter experimental information in the window opened: Genotype (experimental conditions), Cross ID, Fish number, etc. Set the destination folder for data storage. Choose the desired Light-Dark (L/D) cycle from one of the followings: L/D, L/L, or D/D. Adjust daytime light connection/timer accordingly. Use fish size toggle to select thresholds for fish detection: use `Small fish` for larvae younger than 12 dpf and `Big fish` for those that are older. To start recording, click `Select Background` when there's no fish in the FOV.

Larvae older than 5 dpf should be fed every 24 h with 1–2 mL of diluted cultured rotifers. To feed fish, click `Stop program` to stop the current session. Feed with rotifers and allow a pause of 30 min before re-starting the experiment.

At the end of the experiment, click `Stop program` and remove fish from the box. Each session (from `Start` to `Stop program`) generates one `.dlm` data file and a corresponding `.ini` metadata file.

Software requirement for data analysis

To analyze behavior data using code provided, one needs Python 3, analysis scripts, and various Python modules. An integrated development environment (IDE) is recommended to edit, debug, and run the code. If you don't have a personal preference, we recommend using Visual Studio Code (Microsoft). Analysis and visualization code was developed using Python 3. For the ease of package management, we suggest the use of environment management tools, such as miniconda.

The most recent version of the code we use to analyze SAMPL data can be found online at https://. Download the entire directory by pressing the green Code button and downloading the ZIP file (orange box) so that you can make changes as needed for your project. The `src` folder contains all scripts. The `sample figures` folder contains examples of plots from the visualization functions. Please refer to the `README` for instructions and user guides.

To set up a virtual environment, open a new terminal or use the terminal in your IDE, and type:

conda create -n <myenv>

where `<myenv>` is substituted with any desired name for the environment. Next, activate this environment

conda activate <myenv>

and install packages required for analysis and plotting using

conda install <package>

Below is a list of required packages[133–139] other than those included in Python 3.10.4.

- astropy = 5.1
- pandas = 1.4.4
- pytables = 3.7.0
- matplotlib = 3.5.2
- numpy = 1.23.3
- scipy = 1.9.1
- seaborn = 0.12.0
- tqdm = 4.64.1

- scikit-learn = 1.1.1

For a complete list of packages, refer to the `environment.yml` file.

Bout analysis

Analysis and plotting scripts support two types of data structures. The first option is one root directory containing all data files.

```
root
├── data1.dlm
├── data1 parameters.ini
├── data2.dlm
├── data2 parameters.ini
└── ...
```

The second is a root directory containing subfolders with the necessary files indicating experimental repeats.

```
root
├── exp repeat 1
│   ├── data1.dlm
│   ├── data1 parameters.ini
│   ├── data2.dlm
│   ├── data2 parameters.ini
│   └── ...
└── exp repeat 2
    ├── data1.dlm
    ├── data1 parameters.ini
    ├── data2.dlm
    ├── data2 parameters.ini
    └── ...
```

Run the analysis script … `/src/SAMPL_analysis/SAMPL_analysis.py` and input data directory (directory of the root folder) and the frame rate as instructed. This function aligns bouts in `.dlm` files within a directory so that peak speed is at time 0 ms, with 500 ms of activity before and 300ms of activity after. It is important to note that all files in the same subfolders under the input directory will be combined to extract bout parameters. The analysis script will take the submitted directory and analyze all data files within it, including all subfolders in its search, regardless of depth. Subfolders can be used to separate analyses, experimental conditions, or repeats. Data with different frame rates should be analyzed separately to ensure proper parameter calculation, as only one can be used at a time.

The program will skip the current `.dlm` file if it fails to detect a bout in it. However, errors are expected if files contain too little recorded data to extract a bout. Therefore, we suggest removing any `.dlm` files that are smaller than 1 MB.

When analysis is done, it will save three data files (`.h5`), four catalog files (`.csv`), and two metadata files (`.csv`) under the same directory as the data is in. Below is an example of an analyzed directory.

```
root
├── data1.dlm
├── data1 parameters.ini
├── data2.dlm
├── data2 parameters.ini
├── all_data.h5
├── bout_data.h5
├── IEI_data.h5
├── analysis info.csv
├── root metadata.csv
├── catalog all_data.csv
├── catalog bout_data.csv
├── catalog IEI_data.csv
└── data_file_explained.csv
```

Visualizing results

After analysis, the scripts under the `visualization` folder are used to extract swim parameters and kinetics, and visualize them. For more detailes, refer to **Note S4** and the README document. Each function can be run individually and will ask for the directory path to your data (see the **bout analysis** section above). Alternatively, use `plot_all.py` to plot all figures.

If the data size from a single repeat is not adequate for parameter calculation, we suggest combining data from multiple repeats and use sampling techniques such as Jackknifing for error estimation.
